# Mitotic DNA synthesis is caused by transcription-replication conflicts in BRCA2-deficient cells

**DOI:** 10.1016/j.molcel.2022.07.011

**Published:** 2022-09-15

**Authors:** Florian J. Groelly, Rebecca A. Dagg, Michalis Petropoulos, Giacomo G. Rossetti, Birbal Prasad, Andreas Panagopoulos, Teressa Paulsen, Angeliki Karamichali, Samuel E. Jones, Fena Ochs, Vasilis S. Dionellis, Emilia Puig Lombardi, Matthieu J. Miossec, Helen Lockstone, Gaëlle Legube, Andrew N. Blackford, Matthias Altmeyer, Thanos D. Halazonetis, Madalena Tarsounas

**Affiliations:** 1 Genome Stability and Tumourigenesis Group, Department of Oncology, Oxford Institute for Radiation Oncology, University of Oxford, Oxford OX3 7DQ, UK; 2 Department of Molecular Biology, University of Geneva, 1205 Geneva, Switzerland; 3 Department of Molecular Mechanisms of Disease, University of Zurich, 8057 Zurich, Switzerland; 4 Department of Oncology, MRC Weatherall Institute of Molecular Medicine, University of Oxford, John Radcliffe Hospital, Oxford OX3 9DS, UK; 5 Department of Biochemistry, University of Oxford, South Parks Road, Oxford OX1 3QU, UK; 6 Bioinformatics and Statistical Genetics Core, Wellcome Trust Centre for Human Genetics, University of Oxford, Oxford OX3 7BN, UK; 7 LBCMCP, Centre de Biologie Intégrative (CBI), CNRS, Université de Toulouse, UT3, Toulouse 31062, France

**Keywords:** BRCA2, mitotic DNA synthesis, MiDAS, transcription-replication conflicts, TRCs, R-loops, genome stability

## Abstract

Aberrant replication causes cells lacking BRCA2 to enter mitosis with under-replicated DNA, which activates a repair mechanism known as mitotic DNA synthesis (MiDAS). Here, we identify genome-wide the sites where MiDAS reactions occur when BRCA2 is abrogated. High-resolution profiling revealed that these sites are different from MiDAS at aphidicolin-induced common fragile sites in that they map to genomic regions replicating in the early S-phase, which are close to early-firing replication origins, are highly transcribed, and display R-loop-forming potential. Both transcription inhibition in early S-phase and RNaseH1 overexpression reduced MiDAS in BRCA2-deficient cells, indicating that transcription-replication conflicts (TRCs) and R-loops are the source of MiDAS. Importantly, the MiDAS sites identified in BRCA2-deficient cells also represent hotspots for genomic rearrangements in *BRCA2*-mutated breast tumors. Thus, our work provides a mechanism for how tumor-predisposing *BRCA2* inactivation links transcription-induced DNA damage with mitotic DNA repair to fuel the genomic instability characteristic of cancer cells.

## Highlights

• BRCA2 loss triggers mitotic DNA synthesis (MiDAS) at loci that replicate in early S-phase
• Transcription-replication conflicts and R-loops underlie MiDAS activation
• MiDAS sites triggered by BRCA2 loss are distinct from those caused by aphidicolin
• RAD52-mediated MiDAS promotes genome integrity in BRCA2-deficient cells

## Graphical abstract

**Figure:**
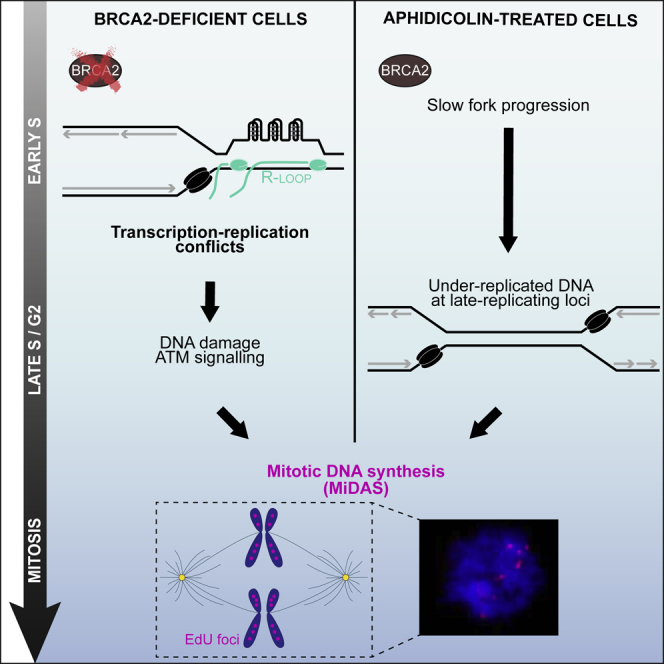

## Teaser

BRCA2-deficient cells activate mitotic DNA synthesis (MiDAS) to complete genome duplication. Groelly et al. show that in the absence of BRCA2, MiDAS originates from DNA lesions caused by transcription-replication conflicts (TRCs) and R-loops during the early S-phase. These sites are therefore distinct from aphidicolin-induced common fragile sites.

## Introduction

Accurate DNA replication is required for genome integrity, cell survival, and disease prevention. Aberrant replication rates and fork stalling at physical barriers obstruct fork progression and lead to the accumulation of DNA damage and genome instability, the underlying cause of numerous pathologies, including cancer (Zeman and Cimprich, 2014). Barriers to replication include DNA secondary structures (such as G-quadruplexes [G4s]), DNA repetitive elements (including minisatellites, rDNA, and telomeres), or sites of transcription-replication conflicts (TRCs) (García-Muse and Aguilera, 2016; Hamperl and Cimprich, 2016).

Transcription and replication share the same DNA template. Consequently, encounters between the replisome and the transcription machinery can lead to TRCs, which represent an endogenous source of replication-associated DNA lesions. If unrepaired, these lesions lead to DNA double-strand breaks (DSBs), mutations, and rearrangements, which cause rampant genomic instability (Hamperl et al., 2017; Lang et al., 2017; Macheret and Halazonetis, 2018; Sankar et al., 2016). TRCs can occur co-directionally, when replication and transcription machineries move in the same direction, or head-on, when they move toward each other.

RNA-DNA hybrids known as R-loops are transient secondary structures that assemble when a nascent transcript invades double-stranded DNA, binding to the template strand and displacing the non-template DNA as a single-stranded D-loop (Roberts and Crothers, 1992; Roy et al., 2010). R-loops form across the genome and are involved in multiple physiological processes, including the regulation of gene expression, transcription termination, and immunoglobulin class-switch recombination (Ginno et al., 2013; Skourti-Stathaki et al., 2011; Yu et al., 2003). Because R-loops interfere with transcription and replication fork progression, they also represent a potent source of DSBs and genomic instability (Chang et al., 2017; Chappidi et al., 2020; Gan et al., 2011). Therefore, cellular R-loop levels are tightly regulated, either by mechanisms preventing their formation or by activities that dismantle R-loop structures after they are formed. For example, RNA-binding proteins such as components of the transcription export 2 (TREX-2) complex act at the interface between transcription and messenger ribonucleoprotein biogenesis and can bind nascent RNA, thereby preventing R-loop formation (Bhatia et al., 2014; Domínguez-Sánchez et al., 2011). Conversely, R-loops can be resolved by helicases such as Sgs1, the yeast ortholog of BLM (Chang et al., 2017), or by the RNA-DNA helicases senataxin (Hatchi et al., 2015; Yüce and West, 2013) and Aquarius (Sollier et al., 2014). The RNase H family of endonucleases also mediate R-loop resolution by cleaving the RNA within RNA-DNA hybrids (Cerritelli and Crouch, 2009). In the absence of any of these mechanisms, cells accumulate R-loops and the ensuing DNA damage contributes to genome instability and other cancer pathologies (Chang et al., 2017; Hatchi et al., 2015; Sollier et al., 2014).

The tumor suppressors BRCA1 and BRCA2 have also been implicated in R-loop metabolism. While BRCA1 interacts with the helicase senataxin to resolve R-loops within transcription termination regions (Hatchi et al., 2015), BRCA2 prevents R-loop accumulation in replicating cells (Bhatia et al., 2014) via mechanisms including recruitment and activation of the RNA helicase DDX5 (Sessa et al., 2021) or negative regulation of RNA polymerase II pausing (Shivji et al., 2018).

BRCA2 has physiological roles in maintaining genome integrity by facilitating DSB repair through homologous recombination (HR), an error-free DNA repair pathway (Li and Heyer, 2008). As BRCA2-deficient cells lack HR (Moynahan et al., 2001), they rely on error-prone DNA repair pathways, most commonly end-joining reactions (Ceccaldi et al., 2015), leading to mutations and chromosome rearrangements. In addition, BRCA2 facilitates DNA replication by protecting stalled forks from nucleolytic degradation (Lemaçon et al., 2017; Ray Chaudhuri et al., 2016; Schlacher et al., 2011; Zimmer et al., 2016). Thus, cells and tumors lacking BRCA2 accumulate spontaneous DSBs and chromosome rearrangements that drive replication-associated genomic instability.

As a result of these replication pathologies, BRCA2-deficient cells enter mitosis with incompletely replicated DNA, displayed as bridges between sister chromatids in anaphase. Failure to resolve these bridges leads to chromosome mis-segregation, 53BP1 nuclear bodies, and micronuclei (Feng and Jasin, 2017; Lai et al., 2017). Our previous work showed that MUS81 and POLD3 are recruited to under-replicated loci upon mitotic entry, where they promote mitotic DNA synthesis (MiDAS), thereby completing genome replication and ensuring correct chromosome segregation (Lai et al., 2017). However, precisely which genomic loci require BRCA2 for their replication and therefore trigger MiDAS when BRCA2 is inactivated remains unknown.

Here, we apply a high-throughput sequencing method for the detection of nascent DNA synthesis in mitosis (Macheret et al., 2020), to precisely map, genome-wide, the sites where MiDAS occurs in cells lacking BRCA2. We find that these sites localize within genes transcribed and replicated during early S-phase and are therefore distinct from the aphidicolin-induced common fragile sites (CFSs). Inhibition of transcription, specifically during early S-phase, reduced the frequency of MiDAS events in BRCA2-deficient cells, indicating that they arose from TRCs. Moreover, these MiDAS sites co-localized with regions prone to form R-loops in unperturbed cells and RNaseH1 overexpression reduced the rate of MiDAS activation. Thus, R-loops trigger MiDAS in the absence of BRCA2. Furthermore, we demonstrate that MiDAS reactions were RAD52-dependent and acted to limit the genomic instability caused by BRCA2 inactivation. Importantly, the MiDAS sites identified in this study in cells lacking BRCA2 are enriched in chromosome rearrangements in *BRCA2*-mutated breast tumors. Taken together, these results indicate that BRCA2 acts in early S-phase to protect against DNA damage induced by TRCs and R-loops, thereby preventing genomic instability and tumorigenesis.

## Results

### Genome-wide mapping of MiDAS sites in BRCA2-deficient cells

**Figure 1:**
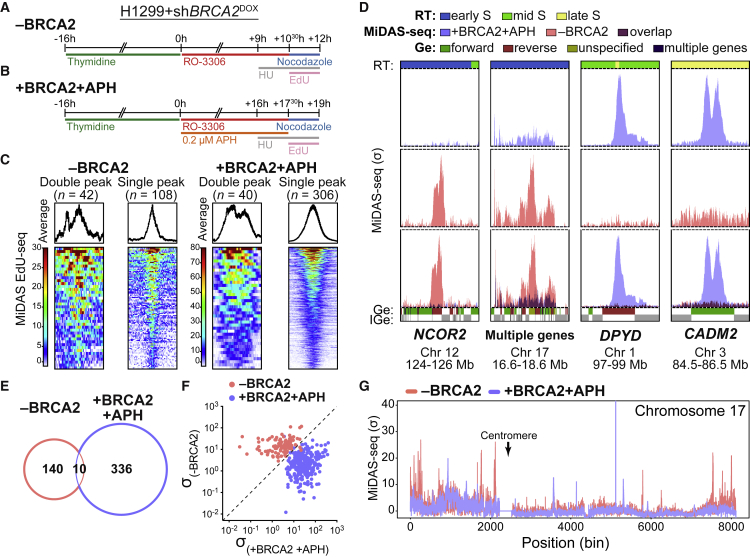
Genome-wide mapping of MiDAS sites in BRCA2-deficient cells (A) Experimental timeline for MiDAS-seq in BRCA2-deficient (−BRCA2) H1299 cells. *BRCA2* shRNA was induced by addition of 2 μg/mL DOX to the growth media 48 h before starting the experiment. (B) Experimental timeline for MiDAS-seq in aphidicolin-treated BRCA2-proficient (+BRCA2 +APH) H1299 cells. (C) Average MiDAS-seq signal across all identified peaks in BRCA2-deficient (−BRCA2; n = 150) and aphidicolin-treated BRCA2-proficient (+BRCA2 +APH; n = 346) H1299 cells. Span of genomic region, 880 kb. (D) MiDAS-seq profiles (σ values) at representative genomic regions for BRCA2-deficient (−BRCA2; pink) and for aphidicolin-treated BRCA2-proficient (+BRCA2 +APH; blue) H1299 cells. RT, replication timing; Ge, genes; IGe, intergenic regions. Bin resolution, 10 kb. (E) Venn diagram of overlapping MiDAS sites (within ±600 kb) between BRCA2-deficient (−BRCA2) and aphidicolin-treated BRCA2-proficient (+BRCA2 +APH) H1299 cells. (F) Scatterplots of MiDAS-seq σ values at all peaks shown in (C). (G) Whole-chromosome view of the MiDAS-seq profiles (σ values) across chromosome 17 for BRCA2-deficient (−BRCA2; pink) and aphidicolin-treated BRCA2-proficient (+BRCA2 +APH; blue) H1299 cells. See also Figures S1–S3 and Tables S1 and S2.

Our published work (Lai et al., 2017) demonstrated that BRCA2-deficient cells progress into mitosis with incompletely replicated DNA. In this case, replication resumes during mitosis in a process known as MiDAS, which can be visualized microscopically through foci marking sites of EdU incorporation in mitotic cells lacking BRCA2. This method, however, could not pinpoint the genomic sites where MiDAS occurs. To map at high-resolution the genomic regions undergoing MiDAS in BRCA2-deficient cells, we established experimental conditions for labeling nascent DNA in mitotic cells. We took advantage of a previously described high-throughput sequencing protocol for MiDAS detection at aphidicolin-induced CFSs in U2OS cells (Macheret et al., 2020), which we optimized for human non-small cell lung carcinoma H1299 cells (Figures 1A and 1B). The H1299 cells used in this study carry a doxycycline (DOX)-inducible short hairpin RNA (shRNA) against *BRCA2* (Zimmer et al., 2016), which enables the effective inhibition of BRCA2 expression after 48 h of DOX treatment (Figure S1A).

To identify the genomic sites undergoing MiDAS upon BRCA2 abrogation, we synchronized cells at the G1/S transition using a single thymidine block and released them in media containing the CDK1 inhibitor RO-3306 (Figure 1A). This treatment caused cell-cycle arrest in G2, reflected by the lack of histone H3 phosphorylation at Ser10 in FACS analyses (Figure S1B). After a 10.5-h incubation with RO-3306, we released the cells into mitosis in the presence of nocodazole and EdU (Figure 1A). Entry into mitosis was demonstrated by FACS analyses of histone H3 Ser10 phosphorylation (Figure S1C), while EdU incorporation was used to monitor MiDAS. To exclusively capture DNA synthesis in mitosis, we inhibited S/G2 replication with hydroxyurea, as previously described (Macheret et al., 2020). EdU-labeled DNA was isolated from mitotic “shake-off” cells and analyzed by high-throughput sequencing for high-resolution mapping of MiDAS sites genome-wide (Macheret et al., 2020). In addition, we established conditions for mapping aphidicolin-induced MiDAS sites in BRCA2-proficient H1299 cells (Figure 1B). These cells were released from G1/S arrest in media containing RO-3306 and low-concentration aphidicolin (0.2 μM) and subsequently processed, as described above, for mitotic cell isolation (Figure S1D). We refer to the protocols used here to identify MiDAS sites as MiDAS-seq, to differentiate them from the EdU-seq protocols used for mapping sites of replication origin firing (Dagg et al., 2021; Macheret and Halazonetis, 2018).

To validate the results obtained by the MiDAS-seq protocol, we compared the MiDAS sites induced by aphidicolin treatment in BRCA2-proficient H1299 cells (Figure 1B) to those induced by the same treatment in U2OS cells (Macheret et al., 2020). There was a 47% overlap among MiDAS regions in the two cell lines (Figures S2A and S2B), which is comparable to the MiDAS site overlap in aphidicolin-treated U2OS and HeLa cells (Macheret et al., 2020). Moreover, as seen previously (Macheret et al., 2020), some large MiDAS regions in aphidicolin-treated H1299 cells contained two peaks of strong EdU incorporation (Figures S2A and S2C), consistent with replication forks being present at both edges of the MiDAS region.

Next, we mapped, genome-wide, the MiDAS sites induced by BRCA2 inactivation and compared them with those induced by aphidicolin treatment in BRCA2-proficient H1299 cells. Applying the MiDAS-seq protocol to H1299 cells treated with DOX (−BRCA2; Figure 1A) identified 150 MiDAS sites (Figure 1C; Table S1), while 346 MiDAS sites (Figure 1C; Table S2) were revealed in H1299 cells treated with aphidicolin (+BRCA2+APH; Figure 1B). The classification of MiDAS events based on sigma values showed a higher incidence of single peaks in both conditions (Figure 1C). When the same protocol was applied to BRCA2-proficient H1299 cells unchallenged by aphidicolin, only 18 MiDAS sites were detected (Figures S3A–S3D), which was consistent with the low frequency of mitotic EdU foci in BRCA2-proficient cells reported in our previous study (Lai et al., 2017). These sites were largely non-overlapping with the MiDAS sites identified in BRCA2-deficient cells (Figures S3C and S3D).

Strikingly, peak analysis showed that the genomic sites undergoing MiDAS in cells lacking BRCA2 (Figure 1D; pink) were distinct from the MiDAS sites identified in aphidicolin-treated cells (Figure 1D; blue). Quantification indicated that only 10 out of the 150 MiDAS sites induced by BRCA2 inactivation overlapped with those induced by aphidicolin in BRCA2-proficient cells (Figure 1E). Scatterplots of the MiDAS-seq signal detected in BRCA2-deficient versus aphidicolin-treated cells further supported the clear distinction between the two types of MiDAS events (Figure 1F). As an example, the MiDAS-seq profile of chromosome 17 illustrates the mutual exclusivity between the MiDAS peaks mapped in the BRCA2-deficient (Figure 1G; pink) and aphidicolin-treated cells (Figure 1G; blue). The observation that MiDAS events triggered by the loss of BRCA2 occur at different genomic sites from those induced by aphidicolin suggests that they arise by different mechanisms.

### MiDAS sites induced by BRCA2 inactivation map to genes within early-replicating genomic regions

MiDAS is defined as the process of DNA repair synthesis occurring in prometaphase at sites susceptible to replication stress, with CFSs as a prominent example (Minocherhomji et al., 2015). CFSs encompass genomic regions that replicate in the late S-phase (Debatisse et al., 2012; Le Beau et al., 1998). Their replication can be further delayed by treatment with aphidicolin, which also triggers MiDAS at these sites (Macheret et al., 2020). The clear distinction observed between MiDAS events in aphidicolin-treated and BRCA2-deficient cells prompted us to determine whether the latter occurred at CFSs. For this purpose, we used the subset of cytogenetically defined CFSs that map to defined genes (n = 73 CFSs spanning 159 genes; Wilson et al., 2015) and found that only 3.1% (4 out of 129) of the genes within MiDAS sites in BRCA2-deficient cells coincided with CFS genes, in contrast to 30% (70 out of 234) of the genes within MiDAS sites in aphidicolin-treated cells (Figure S3E). Thus, MiDAS events triggered by BRCA2 inactivation occur at genomic sites which are distinct from CFSs.

**Figure 2:**
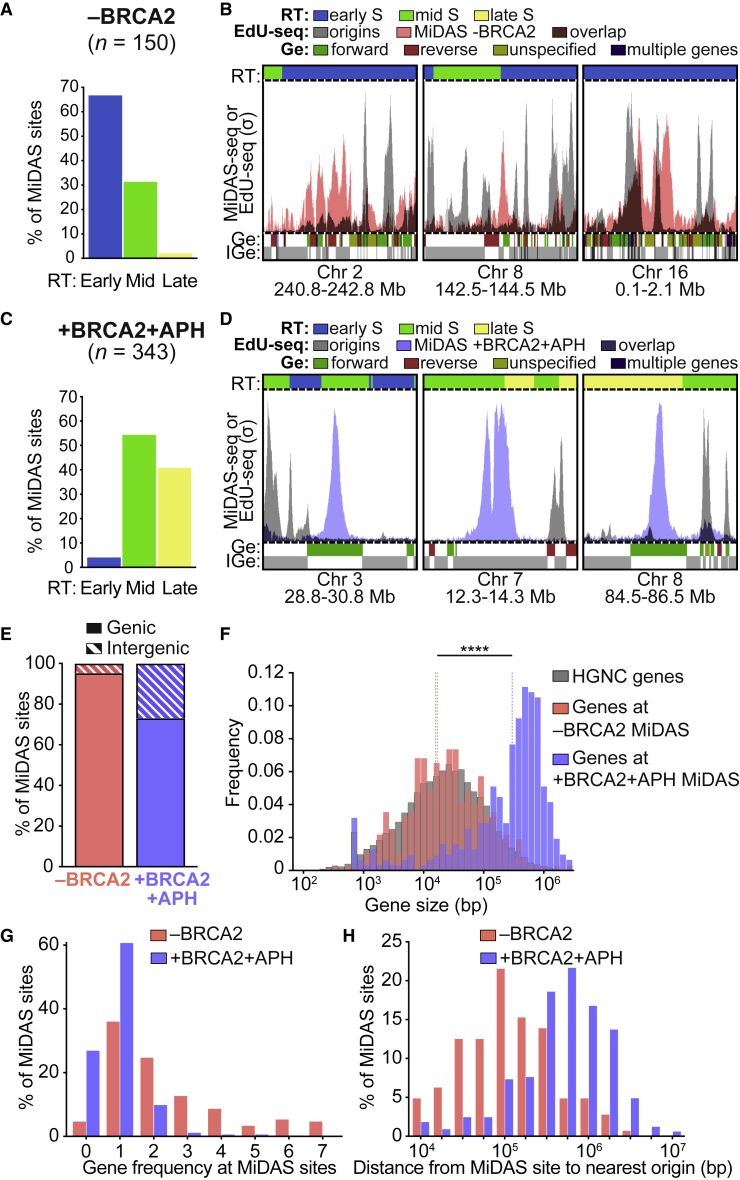
MiDAS sites induced by BRCA2 inactivation map to genes within early-replicating genomic regions (A) Distribution of MiDAS sites (n = 150) identified in BRCA2-deficient (−BRCA2) H1299 cells within genomic regions replicating in early, mid, and late S-phase. (B) MiDAS sites identified using MiDAS-seq (pink) and replication initiation sites identified using EdU-seq (Dagg et al., 2021; gray) at representative genomic regions in BRCA2-deficient (−BRCA2) H1299 cells. RT, replication timing; Ge, genes; IGe, intergenic regions. Bin resolution, 10 kb. (C) Similar to (A) for MiDAS sites (n = 343) identified in aphidicolin-treated BRCA2-proficient (+BRCA2 +APH) H1299 cells. Note: 3 sites have undefined replication timing. (D) Similar to (B) for MiDAS sites identified using MiDAS-seq (blue) in aphidicolin-treated BRCA2-proficient (+BRCA2 +APH) H1299 cells. (E) Distribution of genic and intergenic MiDAS sites in BRCA2-deficient (−BRCA2; n = 150; pink) and in aphidicolin-treated BRCA2-proficient (+BRCA2 +APH; n = 346; blue) H1299 cells. (F) Size frequency distribution of all protein-encoding human genes in HGNC database (gray) and of protein-encoding genes within genic MiDAS sites shown in (E). The analysis includes 367 genes in BRCA2-deficient (−BRCA2; pink) and 314 genes in aphidicolin-treated BRCA2-proficient (+BRCA2 +APH; blue) H1299 cells. Dotted lines indicate median values for each gene set. Statistical significance was determined by a two-tailed Mann-Whitney test; ^∗∗∗∗^p ≤ 0.0001. (G) Gene frequency in the vicinity (±50 kb) of MiDAS site in BRCA2-deficient (−BRCA2; pink) and aphidicolin-treated BRCA2-proficient (+BRCA2 +APH; blue) H1299 cells. (H) Frequency distribution of the distance between each MiDAS site and the nearest origin in BRCA2-deficient (−BRCA2; n = 146; pink) and aphidicolin-treated BRCA2-proficient (+BRCA2 +APH; n = 333; blue) H1299 cells. See also Figure S3.

To elucidate the origin of the MiDAS sites induced by a loss of BRCA2, we first investigated the replication timing (RT) of genomic regions containing MiDAS peaks. Our results revealed that MiDAS events triggered by BRCA2 inactivation map largely to genomic regions replicating in early S-phase (Figures 2A and 2B). This was in stark contrast with the aphidicolin-induced MiDAS events, which map to regions replicating in mid and late S-phase (Figures 2C and 2D), consistent with their propensity to co-localize with CFSs (Macheret et al., 2020).

Next, we characterized the position of MiDAS sites relative to genes and intergenic genomic regions. We found that the majority (95.3%) of MiDAS sites in BRCA2-deficient cells localized within genes, while in aphidicolin-treated cells 73.1% of the MiDAS sites were within genes (Figure 2E). The length of genes containing aphidicolin-induced MiDAS (median 5.6 × 10^5^ bp) significantly exceeds that of genes with MiDAS events induced by BRCA2 abrogation (median 4.3 × 10^4^ bp; Figure 2F), which is the same as the average gene size for the human genome. Interestingly, when we compared gene density around MiDAS peaks (±50 kb), we observed higher gene frequencies around those in BRCA2-deficient cells than those in aphidicolin-treated cells (Figure 2G). Thus, MiDAS events in cells lacking BRCA2 occur in average size genes, localized in gene-rich regions of the genome.

A common feature of CFSs and aphidicolin-induced MiDAS sites is the scarcity of replication origins in their genomic environment (Le Tallec et al., 2011; Letessier et al., 2011; Macheret et al., 2020). To test whether this was also the case for MiDAS events triggered by BRCA2 inactivation, we calculated the distance between each MiDAS peak and the nearest replication origin, previously mapped by EdU-seq (Dagg et al., 2021). We found that the MiDAS sites in BRCA2-deficient cells were positioned closer to replication origins than the aphidicolin-induced MiDAS sites (Figure 2H). The positions of replication origins relative to MiDAS sites are illustrated in the images of representative genomic loci for each condition (Figures 2B and 2D).

Taken together, these results demonstrate that BRCA2 abrogation and aphidicolin treatment induce MiDAS at spatially distinct genomic loci, which differ in their RT, gene density, and proximity to replication origins. Consequently, each of the two types of MiDAS events is likely to be triggered by distinct replication pathologies.

### Genes within MiDAS sites are transcribed in early S-phase

**Figure 3:**
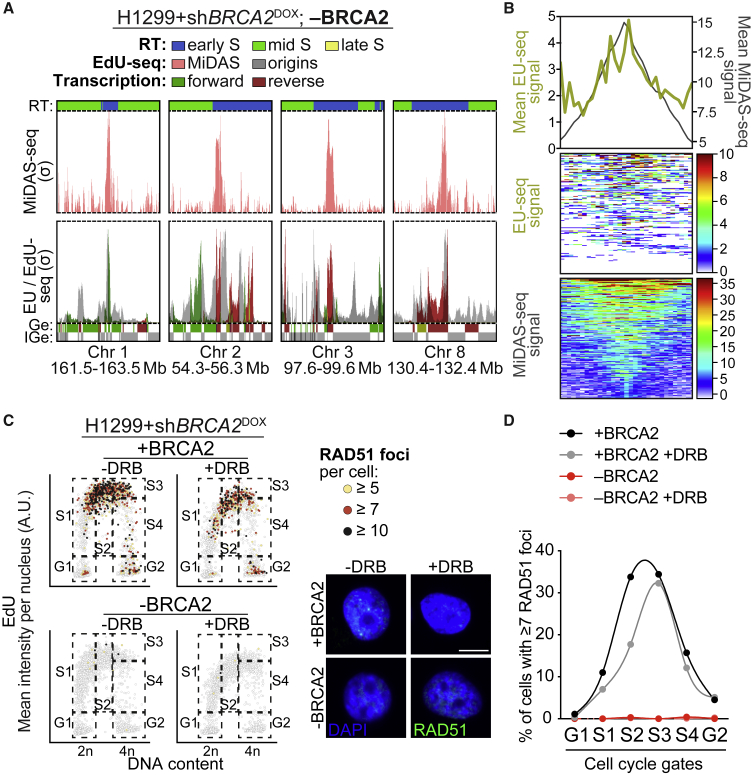
Genes within MiDAS sites are transcribed in early S-phase (A) Top row: MiDAS-seq profiles (σ value) at representative genomic regions in BRCA2-deficient (−BRCA2) H1299 cells. Bottom row: EU-seq profiles (σ value) for nascent transcription at the same genomic sites, detected 100 min after release from single thymidine block (green, forward direction of transcription; red, reverse). Replication initiation profiles are shown in gray. RT, replication timing; Ge, genes; IGe, intergenic regions. Bin resolution, 10 kb. (B) Average EU-seq signal for nascent transcription (yellow) and MiDAS-seq signal (black), centered across MiDAS peaks (n = 150) identified in BRCA2-deficient (−BRCA2) H1299 cells. Span of genomic region, 200 kb. (C) BRCA2-proficient (+BRCA2) and -deficient (−BRCA2) H1299 cells were treated with 75 μM DRB for 4 h, pulse-labeled with EdU for 30 min. QIBC scatterplots of DNA content versus EdU mean intensity show RAD51 foci number in color scale. Boxes indicate cell populations used for quantification. Representative images of RAD51 foci (green) and DAPI staining (blue) for the 4 conditions are shown. DAPI, 4′,6-diamidino-2-phenylindole. Scale bars, 10 μm. (D) Quantification of cells treated as in (C) and having ≥7 RAD51 foci per cell. Graph shows data from one experiment representative of n = 2 independent experiments.

Our observation that MiDAS peaks induced by BRCA2 abrogation localize within gene-rich regions and near replication origins supports the concept that these MiDAS events likely stem from conflicts between transcription and replication. Although RT analyses showed that the MiDAS sites replicate in early S-phase (Figures 2A and 2B), it was unknown whether the genes within MiDAS sites were also transcribed in early S-phase. To address this, we performed EU-seq assays for gene expression (Macheret and Halazonetis, 2018) in BRCA2-deficient cells collected 100 min after release from thymidine-induced G1/S arrest. EU was added to the media 20 min before cell collection to enable the capturing of nascent early S-phase transcripts (Figure 3A). Combined MiDAS-seq and EU-seq profiles showed a substantial overlap between MiDAS peaks (Figure 3A; pink) and peaks of EU incorporation in early S-phase (Figure 3A; green and red, depicting forward and reverse transcription, respectively). Quantification revealed that 80% (120 out of 150) of the MiDAS sites in BRCA2-deficient cells were transcribed in early S-phase. The early replication origins firing in the vicinity of MiDAS peaks (Figure 3A; gray) were positioned in non-transcribed regions adjacent to actively transcribed genes, as previously reported (Macheret and Halazonetis, 2018; Petryk et al., 2016). The average EU-seq signal around MiDAS sites (±100 kb) further confirmed that these sites were actively transcribed (Figure 3B). Collectively, these results demonstrate that in BRCA2-deficient cells MiDAS events map to regions that are replicated and transcribed in early S-phase and that may represent sites of TRCs.

Our results also suggested the possibility that BRCA2, possibly together with other HR factors (e.g., RAD51), help to resolve TRCs that occur in early S-phase. Quantitative image-based cytometry (QIBC; Ochs et al., 2016; Teloni et al., 2019; Toledo et al., 2013) enabled us to monitor the cell-cycle distribution of spontaneous RAD51 foci in the presence or absence of active transcription (Figures 3C and 3D). As expected, RAD51 foci were not visible in BRCA2-deficient cells but were readily detected in BRCA2-proficient cells, predominantly in S-phase. Treatment with the transcription elongation inhibitor 5,6-dichloro-1-β-D-ribofuranosylbenzimidazole (DRB) caused a visible reduction in the overall frequency of cells with RAD51 foci and markedly affected the early S-phase cells with ≥7 RAD51 foci (Figure 3D). These results indicate that a subset of the spontaneous RAD51 foci forming in S-phase is caused by active transcription.

### MiDAS events in BRCA2-deficient cells are dependent on early S-phase transcription

**Figure 4:**
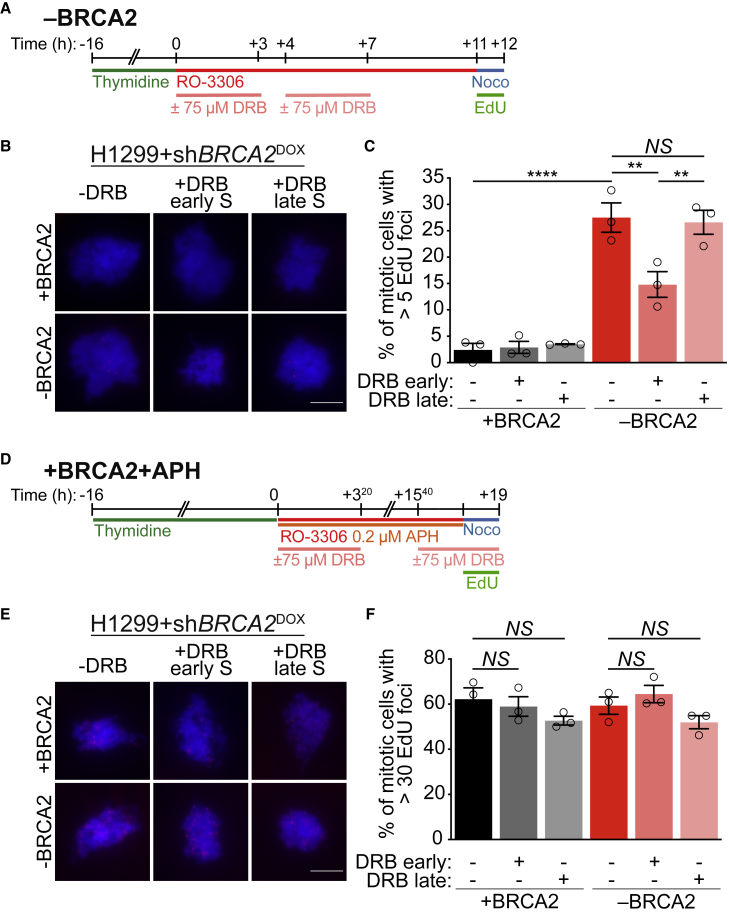
MiDAS events in BRCA2-deficient cells are dependent on early S-phase transcription (A) Experimental timeline for detection of mitotic EdU foci in BRCA2-proficient (+BRCA2) or deficient (−BRCA2) H1299 cells. *BRCA2* shRNA was induced by the addition of 2 μg/mL DOX to the growth media 48 h before starting the experiment. (B) Representative images of cells treated as in (A). Scale bars, 10 μm. (C) Quantification of mitotic cells treated as in (A) and having >5 EdU foci per cell. Graph and error bars represent the mean and SEM of n = 3 independent experiments. A minimum of 50 cells were analyzed per condition per experiment. (D) Experimental timeline for detection of mitotic EdU foci in aphidicolin-treated BRCA2-proficient (+BRCA2+APH) or -deficient (−BRCA2+APH) H1299 cells. *BRCA2* shRNA was induced by the addition of 2 μg/mL DOX to the growth media 48 h before starting the experiment. (E) Representative images of cells treated as in (D). Scale bars, 10 μM. (F) Quantification of EdU foci per mitotic cell treated as in (D) >30 foci per cell. Graph and error bars represent the mean and SEM of n = 3 independent experiments. A minimum of 50 cells were analyzed per condition per experiment. p values were calculated using one-way ANOVA followed by a Tukey test. ^∗∗^p ≤ 0.01; ^∗∗∗∗^p ≤ 0.0001; NS, p > 0.05. See also Figure S3.

The position of MiDAS sites near early-firing replication origins and within actively transcribed genes suggested that MiDAS originates at TRCs arising in early S-phase. To further investigate this possibility, we evaluated the impact of inhibiting S-phase transcription on MiDAS in BRCA2-deficient cells. Thus, we released cells from thymidine-induced G1/S arrest into S-phase and used DRB to inhibit transcription, either at early or late stages of S-phase progression (Figure 4A). Mitotic EdU foci, which identify MiDAS events, were detected using conditions similar to those described for MiDAS-seq (Figure 4A). Consistent with our previous work (Lai et al., 2017), BRCA2-deficiency increased the frequency of mitotic cells with EdU foci (Figures 4B and 4C). Strikingly, DRB addition during early S-phase significantly decreased the frequency of EdU-positive BRCA2-deficient mitotic cells, whereas DRB addition during late S-phase had no effect (Figure 4C). The requirement for early S-phase transcription in triggering MiDAS was recapitulated in HeLa cells, where DRB addition during early S-phase abrogated the accumulation of mitotic EdU foci caused by small interfering RNA (siRNA)-mediated BRCA2 depletion (Figure S3F). These results demonstrate that MiDAS events in BRCA2-deficient cells are caused by active transcription in early S-phase.

To test whether the impact of transcription on MiDAS was specific to cells lacking BRCA2, we quantified mitotic EdU foci in aphidicolin-treated cells that had been exposed to DRB either in early or late S-phase (Figure 4D). Consistent with our own results (Figures 1C–1G) and previous reports (Macheret et al., 2020; Minocherhomji et al., 2015), aphidicolin treatment triggered an increase in the frequency of mitotic cells with EdU foci (Figures 4E and 4F) relative to untreated cells (Figures 4B and 4C). Inhibition of transcription with DRB during early or late S-phase did not affect the frequency of EdU-positive aphidicolin-treated mitotic cells (Figure 4F). Thus, early S-phase transcription triggers MiDAS in BRCA2-deficient cells, but not in cells treated with aphidicolin.

### MiDAS events induced by BRCA2 inactivation map to regions of R-loop formation

**Figure 5:**
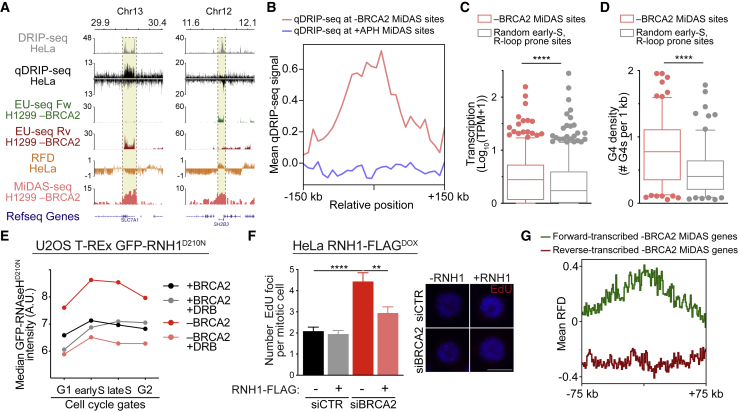
MiDAS events induced by BRCA2 inactivation map to regions of R-loop formation (A) Representative genomic regions of DRIP-seq and qDRIP-seq analyses performed in asynchronous HeLa cells (Crossley et al., 2020; Hamperl et al., 2017), RFD analysis performed in HeLa cells (Petryk et al., 2016), and EU-seq and MiDAS-seq analysis performed in BRCA2-deficient (−BRCA2) H1299 cells. EU-seq was performed 100 min after release from thymidine block. (B) Average qDRIP-seq signal centered across MiDAS peaks identified in BRCA2-deficient (n = 150; −BRCA2; pink) or aphidicolin-treated BRCA2-proficient (n = 346; +BRCA2 +APH; blue) H1299 cells. Span of genomic region, 300 kb. (C) Transcript levels measured by EU-seq 100 min after release from single thymidine block in BRCA2-deficient (−BRCA2) H1299 cells for genes found in the vicinity (±50 kb) of MiDAS sites identified in BRCA2-deficient (−BRCA2) cells or at 150 randomly selected early-replicating, R-loop-prone sites. (D) G-quadruplex (G4) density at MiDAS sites identified in BRCA2-deficient (−BRCA2) cells or at 150 randomly selected early-replicating, R-loop-prone sites. (E) Quantification of RNA-DNA hybrids measured by QIBC of mean chromatin-bound GFP-RNaseH1^D210N^ signal. Graph shows the median values obtained for each of the indicated cell-cycle stages and is representative of n = 3 independent experiments. (F) Quantification of mitotic EdU foci in HeLa expressing a DOX-inducible, FLAG-tagged RNaseH1 (RNH1-FLAG), treated as indicated and collected 9 h after release from single thymidine block with 20 mM EdU added during the final hour. Graph and error bars represent the mean and SEM of a total of 156 mitotic cells per conditions from n = 3 independent experiments. Representative images are shown. Scale bars, 16 μm. (G) Replication fork directionality (RFD) measured by OK-seq (Petryk et al., 2016) at MiDAS sites identified in BRCA2-deficient H1299 cells, which contain a single gene. 22 genes transcribed in the forward direction (green) and 32 genes transcribed in the reverse direction (red) were analyzed. Span of genomic region, 150 kb. p values were calculated using an unpaired two-tailed t test (C and D) or using one-way ANOVA followed by a Tukey test (F). ^∗∗^p ≤ 0.01; ^∗∗∗∗^p ≤ 0.0001. See also Figures S4 and S5 and Tables S3 and S4.

RNA-DNA hybrids form spontaneously during transcription (García-Muse and Aguilera, 2019). Due to their potential to obstruct fork progression, they can represent a major cause of TRCs (Hamperl and Cimprich, 2016). In BRCA2-deficient cells, MiDAS occurs at sites that are concomitantly transcribed and replicated, where R-loops might form. Therefore, we compared the position of MiDAS sites identified in our study with that of R-loops previously mapped genome-wide in unperturbed HeLa cells by DNA-RNA immunoprecipitation sequencing (DRIP-seq; Hamperl et al., 2017) or strand-specific quantitative differential DRIP sequencing (qDRIP-seq; Crossley et al., 2020). Strikingly, we found that a substantial fraction of MiDAS events triggered by a loss of BRCA2 mapped to sites with R-loop forming potential (Figure 5A), while aphidicolin-induced MiDAS sites showed no co-localization with R-loops (Figure S4A). To further investigate a possible link between R-loops and MiDAS in BRCA2-deficient cells, we plotted the mean qDRIP-seq signal across the MiDAS sites (Figure 5B). We observed that the average qDRIP signal peaked at the center of the MiDAS sites identified in BRCA2-deficient cells, whereas aphidicolin-induced MiDAS sites did not show detectable qDRIP-seq signal.

Quantification of DRIP-seq data revealed that 80% of the MiDAS events in BRCA2-deficient cells coincided with R-loops (Table S3), prompting the question of how these R-loops differ from all other R-loops formed genome-wide. A comparison with the peaks of nascent transcription (EU-seq) revealed that the sites where MiDAS co-localized with R-loops were highly transcribed in early S-phase in BRCA2-deficient H1299 cells (Figures 5A and S4B), suggesting that elevated transcription could mediate MiDAS at these sites. To test this possibility, we compared the 150 MiDAS sites induced by BRCA2 abrogation to a set of 150 randomly selected genomic sites, which replicate in early S-phase and are prone to R-loop formation (Table S4). Quantification of EU-seq data obtained from BRCA2-deficient H1299 cells released from thymidine block into early S-phase revealed that the genes within MiDAS sites exhibited significantly higher levels of transcription than the randomly selected genes (Figure 5C). Moreover, we tested whether the differences between the two gene sets extended to their propensity to form G4s. G4s form on guanine-rich single-stranded DNA displaced during DNA replication (Lipps and Rhodes, 2009) or at R-loops. In the latter scenario, G4s are thought to stabilize the R-loops and further impede replication fork progression (Brickner et al., 2022; De Magis et al., 2019; Hamperl and Cimprich, 2014). Therefore, we investigated whether G4s are enriched at the MiDAS sites that coincide with R-loops. We used the genomic location of G4s mapped in primary human B lymphocytes (Chambers et al., 2015) and found a striking increase in G4 density at MiDAS sites in BRCA2-deficient cells compared with randomly selected control sites (Figure 5D). These results show that MiDAS events triggered by BRCA2 inactivation map to genomic regions that can assemble R-loops, which are also highly transcribed and have a high G4 forming potential.

To further probe the association of R-loops with MiDAS induced by BRCA2 deficiency, we monitored R-loop formation in cells in which BRCA2 was depleted using siRNA. For this purpose, we used U2OS cells with DOX-inducible expression of a catalytically inactive, GFP-tagged RNaseH1^D210N^ mutant, which can bind R-loops, but not cleave them (Crossley et al., 2021; Teloni et al., 2019). We documented that the addition of DOX induced the expression of GFP-RNaseH1^D210N^ and that BRCA2 depletion suppressed RAD51 foci formation (Figures S5A–S5C). Quantification of chromatin-bound GFP-RNaseH1^D210N^ (Figures 5E and S5D) demonstrated that BRCA2 depletion increased R-loop levels, consistent with previous observations (Bhatia et al., 2014). Importantly, inhibition of transcription elongation using DRB rescued R-loop accumulation, demonstrating that R-loop formation in BRCA2-deficient cells is transcription-dependent (Figures 5E and S5D).

To determine whether MiDAS in BRCA2-deficient cells depends on R-loops, we monitored the formation of mitotic EdU foci in HeLa cells in which R-loops were degraded using DOX-inducible expression of FLAG-tagged RNaseH1 (RNH1-FLAG; Sollier et al., 2014). Although RNaseH1 overexpression did not affect MiDAS events in cells transfected with control siRNA, it significantly decreased the number of mitotic EdU foci in BRCA2-depleted cells (Figures 5F and S5E), supporting the concept that R-loop accumulation is required for MiDAS activation in cells lacking BRCA2.

### Transcription and replication fork directionality at MiDAS sites

TRCs can be either co-directional or head-on orientation, depending on whether the replication fork and the transcription elongation complex travel in the same or opposite direction, respectively (Hamperl et al., 2017). To assess the impact of TRC orientation on MiDAS caused by BRCA2 deficiency, we retrieved replication fork directionality (RFD) profiles generated by Okazaki fragment sequencing (OK-seq) in asynchronous HeLa cells (Petryk et al., 2016). We found that replication and transcription were largely co-directional at discrete MiDAS sites (Figure 5A), as indicated by negative RFD values (Figure 5A; orange) across the genes transcribed in the reverse direction (Figure 5A, red), and positive RFD values across forward-transcribed genes (Figure 5A, green).

We then focused on the subset of MiDAS peaks which contain a single gene and classified them depending on the direction of transcription (Figure 5G). This approach allowed us to unambiguously compare RFD and transcription orientation at a subset of 54 MiDAS sites in BRCA2-deficient cells. We found that the mean RFD values were positive at MiDAS sites mapping to genes transcribed in the forward direction (Figure 5G, green) and negative at MiDAS genes transcribed in the reverse direction (Figure 5G, red), indicating that replication and transcription are mostly co-directional at these sites. Analysis of the replication and transcription orientation at MiDAS sites transcribed in early S-phase showed co-directionality at ∼62% (74 out of 120) of these sites, while head-on orientation was only observed at ∼18% (21 out of 120) of these sites. The prevalence of co-directional replication and transcription at MiDAS sites is also illustrated at other genomic loci (Figure S4B). Taken together, these results suggest that co-directional, and to a lesser extend head-on, TRCs trigger MiDAS in BRCA2-deficient cells.

Co-directional TRCs associated with sites of R-loop formation were shown to activate ataxia telangiectasia mutated (ATM) (Hamperl et al., 2017), the main checkpoint kinase orchestrating the cellular response to DSBs (Blackford and Jackson, 2017). To test whether ATM activation is required for the repair of TRC-induced DNA damage by MiDAS, we monitored mitotic EdU foci formation in HeLa cells transfected with control or BRCA2 siRNA and treated with ATM inhibitor after release from G1/S arrest. ATM inhibition led to a substantial reduction of MiDAS foci in BRCA2-deficient cells (Figures S6A and S6B). This observation not only corroborated the results of Hamperl et al. (2017), that co-directional R-loops trigger ATM activation, but also suggested that ATM signaling promotes MiDAS at sites of TRC in cells lacking BRCA2.

**Figure 6:**
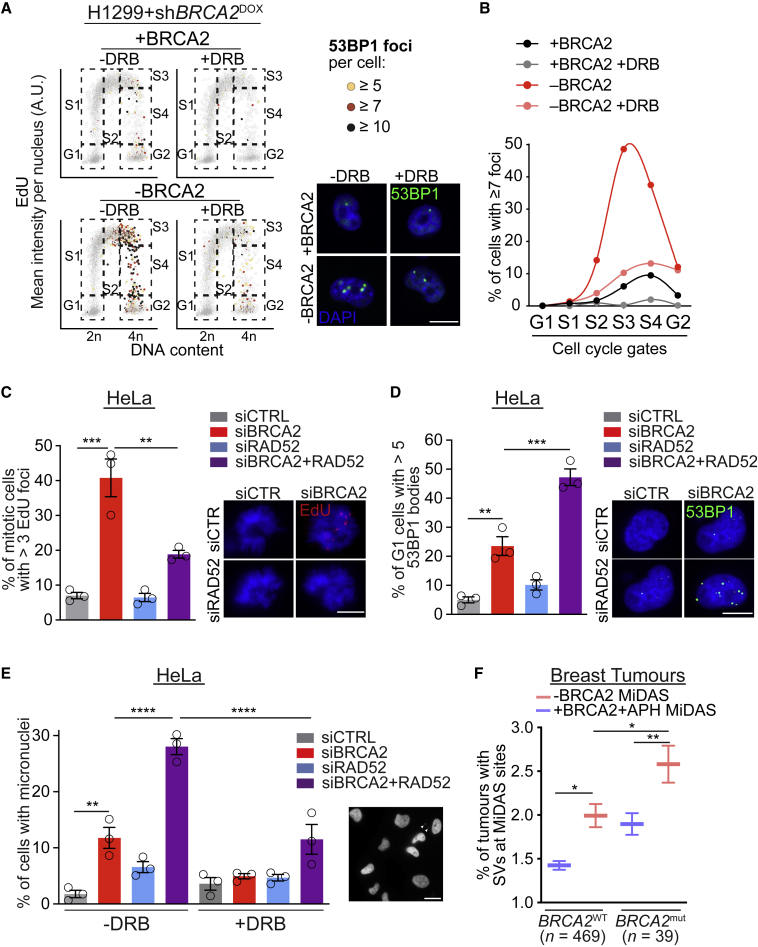
RAD52 is required for MiDAS reactions that preserve the genomic integrity of BRCA2-deficient cells (A) BRCA2-proficient (+BRCA2) and -deficient (−BRCA2) H1299 cells were treated with 75 μM DRB for 4 h, pulse-labeled with EdU for 30 min. QIBC scatterplots of DNA content versus EdU mean intensity show 53BP1 foci number in color scale. Boxes indicate cell populations used for quantification. Representative images of 53BP1 foci (green) and DAPI staining (blue) for the 4 conditions are shown. Scale bars, 10 μm. (B) Quantification of cells treated as in (A) and having ≥7 53BP1 foci per cell. Graph shows data from one experiment representative of n = 3 independent experiments. (C) Quantification of mitotic cells with >3 EdU foci collected 9 h after release from single thymidine block. Graph and error bars represent the mean and SEM of n = 3 independent experiments. A minimum of 50 cells were analyzed per condition per experiment. Representative images are shown. Scale bars, 7 μm. (D) Quantification of G1 cells with >5 53BP1 nuclear bodies collected 10 h after release from single thymidine block. Graph and error bars represent the mean and SEM of n = 3 independent experiments. A minimum of 60 cells were analyzed per condition per experiment. Representative images are shown. Scale bars, 7 μm. (E) Quantification of G1 cells with micronuclei collected 10 h after release from single thymidine block and treated or not with DRB during the first 100 min. Graph and error bars represent the mean and SEM of n = 3 independent experiments. A minimum of 90 cells were analyzed per condition per experiment. (F) Percentage of *BRCA2*-wild-type (n = 469) and *BRCA2*-mutated (n = 39) breast tumors (Nik-Zainal et al., 2016) containing structural variants (SVs) at individual MiDAS sites. A total of n = 150 MiDAS sites identified in BRCA2-deficient (−BRCA2) H1299 cells and of n = 346 MiDAS sites identified in aphidicolin-treated BRCA2-proficient (+BRCA2 +APH) H1299 cells were analyzed. p values were calculated using one-way ANOVA followed by a Tukey test. ^∗^p ≤ 0.05; ^∗∗^p ≤ 0.01; ^∗∗∗^p ≤ 0.001; ^∗∗∗∗^p ≤ 0.0001. See also Figure S6.

Given that ATM activation requires DSB formation, we examined whether BRCA2 deficiency leads to spontaneous DNA damage in S-phase and, based on the results presented so far, whether such damage is dependent on transcription. QIBC analysis of BRCA2-proficient and -deficient cells revealed an accumulation of 53BP1 foci in response to BRCA2-deficiency, specifically in S-phase cells (Figures 6A and 6B). This effect was reversed by inhibiting transcription elongation with DRB. Similar results were obtained in HeLa cells synchronized at the G1/S boundary and released in S-phase in the presence or absence of DRB (Figure S6C). In this setting, siRNA-mediated BRCA2 depletion triggered increased frequencies of early S-phase cells with 53BP1 or γH2AX foci, both abrogated by DRB treatment (Figures S6D and S6E). These results indicate that transcription during S-phase, which can lead to TRCs, is the cause of spontaneous DSBs in BRCA2-deficient cells, a subset of which activate ATM and can be repaired by MiDAS.

### RAD52 is required for MiDAS reactions that preserve genomic integrity in BRCA2-deficient cells

Next, we investigated the molecular requirements for the MiDAS reactions detected in BRCA2-deficient cells. RAD52, a protein acting in DNA repair pathways, including HR (Bai and Symington, 1996), single-strand annealing (SSA; Ochs et al., 2016), and break-induced replication (BIR; Anand et al., 2013; Sotiriou et al., 2016), has been shown to promote MiDAS at sites affected by replication stress induced by aphidicolin (Bhowmick et al., 2016). To test whether RAD52 is similarly required for MiDAS events triggered by BRCA2 inactivation, we depleted BRCA2 and RAD52 in HeLa cells and monitored the impact of their inactivation on mitotic EdU foci formation. Co-depletion of RAD52 together with BRCA2 significantly reduced the frequency of cells exhibiting mitotic EdU foci (Figure 6C). Similar results were obtained using isogenic *RAD52*^*+/+*^ and *RAD52*^*−/−*^ U2OS cells in which BRCA2 was depleted with siRNA (Figure S6F). These results demonstrated that RAD52 is required for the MiDAS reactions in BRCA2-deficient cells.

53BP1 nuclear bodies detectable in G1 mark DNA lesions transmitted to daughter cells upon mitotic chromosome segregation (Harrigan et al., 2011; Lukas et al., 2011; Spies et al., 2019). Because MiDAS resolves DNA damage present in mitotic cells, we investigated whether suppressing MiDAS in BRCA2-deficient cells by depleting RAD52 would lead to increased DNA damage in G1-phase of the next cell cycle. Indeed, BRCA2 depletion increased the frequency of 53BP1 nuclear bodies in HeLa and U2OS cells, and the inactivation of RAD52 led to an even further increase in G1 53BP1 nuclear bodies (Figures 6D and S6G). To evaluate more directly the genomic instability caused by RAD52 inactivation in BRCA2-deficient cells, we examined micronuclei formation. The frequency of micronuclei in BRCA2-depleted HeLa cells was significantly augmented by RAD52 co-depletion (from 12% to 28%; Figure 6E). Importantly, DRB addition during early S-phase markedly reduced micronuclei accumulation (Figure 6E). Together, these results suggest that early S-phase transcription in BRCA2-deficient cells inflicts DNA damage that requires RAD52-dependent MiDAS to be resolved. Thus, MiDAS provides a mechanism that limits the DNA damage and genomic instability driven by BRCA2 abrogation.

It is now well established that TRCs and R-loops represent a potent source of genomic instability, linking transcription to mutations that can cause multiple human pathologies, including cancer (Crossley et al., 2019; Groh et al., 2014). The co-localization of MiDAS sites identified in BRCA2-deficient cells with R-loops and their dependence on TRCs led us to investigate whether these sites are prone to chromosome rearrangements in cancers. We therefore interrogated the whole-genome sequencing data from 560 breast cancers (Nik-Zainal et al., 2016) to determine the rearrangement frequency at the genomic loci that require MiDAS repair upon BRCA2 abrogation or aphidicolin treatment (Figure 1C). We analyzed separately the frequency of rearrangements at these sites in *BRCA2*-wild-type (n = 469) or *BRCA2*-mutated (n = 39) tumors. We found that all breast cancers, independent of their *BRCA2* status, exhibited more frequent genomic rearrangements at MiDAS sites triggered by BRCA2 inactivation than those triggered by aphidicolin treatment (Figure 6F; Tables S1 and S2), suggesting that the former could represent generic hotspots for chromosome breakage in breast tumors. Moreover, frequency of rearrangements at MiDAS sites identified in BRCA2-deficient cells was markedly higher in the tumors with *BRCA2* mutations than the tumors with wild-type *BRCA2* (Figure 6F), supporting the premise that TRCs and R-loops drive genomic instability in human cancers lacking HR.

## Discussion

BRCA2-deficient cells are known to enter mitosis with under-replicated DNA, which activates mitotic DNA repair synthesis (Lai et al., 2017), also known as MiDAS. In this study, we used MiDAS-seq, a technique that combines EdU incorporation in mitotic prophase cells with high-throughput sequencing, to identify the precise genomic sites where MiDAS occurs in BRCA2-deficient cells. Analysis of the MiDAS-seq profiles remarkably demonstrated that these sites are distinct from those induced by aphidicolin (Macheret et al., 2020) and, therefore, also distinct from CFSs. We showed that MiDAS events triggered by BRCA2 inactivation depend on transcription and map to genomic regions with R-loop-forming potential, which are transcribed and replicated in early S-phase. These surprising results indicate that transcription-driven lesions persist through S-phase and are repaired upon onset of mitosis in cells lacking BRCA2. Importantly, we found that these sites are susceptible to chromosome rearrangements in *BRCA2*-mutated breast tumors, suggesting that genomic instability in tumors with compromised BRCA2 function is due in part to pathologies linking transcription with replication and mitotic DNA repair. Therefore, our work provides insights into the origins of cancer in patients carrying *BRCA2* mutations.

### Comparison between MiDAS induced by BRCA2 inactivation and MiDAS induced by aphidicolin treatment

In contrast to MiDAS events induced in human cells by aphidicolin treatment, which map to genomic regions replicating in mid and late S-phase and encompass most known CFSs (Macheret et al., 2020), the MiDAS sites identified in BRCA2-deficient cells co-localize to loci that replicate in early S-phase.

Aphidicolin-induced MiDAS events occur at sites susceptible to replication stress during S-phase, the best-characterized being CFSs (Glover et al., 2017). CFSs correspond to late-replicating regions of the genome (Debatisse and Rosselli, 2019; Le Beau et al., 1998; Letessier et al., 2011; Macheret et al., 2020) where, due to scarcity of replication origins, converging replication forks travel long distances, often causing CFSs to remain under-replicated as cells enter mitosis. Because aphidicolin slows down fork progression throughout S-phase (via inhibition of DNA polymerases; Glover et al., 1984), this effect is further exacerbated at late-replicating regions, which fail to complete replication before mitosis onset and become sites of MiDAS repair. In contrast, early-replicating regions have sufficient time to complete replication during S-phase and, consequently, are unlikely to require repair in mitosis.

BRCA2-deficient cells also display low rates of fork progression (Lai et al., 2017). Paradoxically, in these cells MiDAS events affect early-replicating regions. In other words, sites that initiate replication in early S-phase fail to complete replication and undergo repair synthesis in mitosis, indicating that they arise via distinct mechanisms from the aphidicolin-induced MiDAS events. Further characterization of these sites in cells lacking BRCA2 showed that they are actively transcribed and have R-loop forming potential, suggesting that collisions between transcription and replication make a major contribution to MiDAS events in BRCA2-deficient cells. Consistent with this model, a recent study (Shao et al., 2020) showed that BRCA2 is recruited to the chromatin at TRC sites. Moreover, R-loop formation at these sites was critical, as RNaseH1 overexpression abrogated recruitment.

It is intriguing that collisions between replication and transcription that conceivably occur in late S-phase do not cause MiDAS. In stimulated mouse primary B cells, TRCs map mainly to early-replicating genomic regions (St Germain et al., 2022). This result lends support to our observation that TRC-driven MiDAS events are enriched in early-replicating regions in BRCA2-deficient cells.

### Nature of MiDAS substrates and why they persist from early S-phase into mitosis

In this study, we demonstrate that in cells lacking BRCA2, DNA lesions arise at sites undergoing concomitant transcription and replication in early S-phase, a subset of which is repaired in mitosis via MiDAS reactions. The MiDAS substrate is therefore a TRC-derived structure: either a fork stalled at an R-loop site or a DSB resulting from its collapse. Supporting the former possibility is our result that MiDAS occurs at R-loop sites enriched in G4s and highly transcribed, both leading to R-loop stabilization and fork stalling (Brickner et al., 2022). Supporting the latter is our result that ATM signaling is required for MiDAS. ATM activation is triggered not only by DSBs, which accumulate spontaneously in BRCA2-deficient cells in a transcription-dependent manner, but also by co-transcriptional R-loops (Hamperl et al., 2017), which predominantly co-localize with MiDAS sites in BRCA2-deficient cells. Moreover, previous work using separation-of-function BRCA2 mutants revealed that the loss of HR repair function, but not of fork protection, triggered MiDAS (Feng and Jasin, 2017), further supporting DSBs as the MiDAS substrates. Whether DSBs arising at sites of TRCs and repaired by MiDAS originate from the collapse of forks stalled at R-loop sites is a third possibility that deserves further investigation.

Equally intriguing is the observation that these transcription-induced DSBs persist until mitosis onset in BRCA2-deficient cells. One possible explanation is that DSB repair during S-phase is largely ineffective due to the abrogation of HR repair. Consistent with this, intra-genic DSBs were shown to engage primarily in HR-mediated repair reactions (Aymard et al., 2017; Ouyang et al., 2021). Moreover, one-ended DSBs (such as those initially arising at TRC-stalled forks) activate ATM, which in turn can prevent their repair via end-joining reactions (Balmus et al., 2019).

Nevertheless, MiDAS reactions acted to preserve genomic stability in BRCA2-deficient cells because their abrogation via RAD52 inactivation augmented DNA damage. This was dependent on transcription, further supporting the MiDAS role in repairing TRC-induced lesions. Thus, RAD52-dependent MiDAS activation limits genomic instability in cells lacking BRCA2 and could explain the synthetical lethal interaction between *RAD52* and *BRCA2* gene deletion (Chandramouly et al., 2015; Feng et al., 2011).

### Limitations of the study

In this study, we show that MiDAS provides a mechanism for repair of a subset of the DNA lesions arising spontaneously in cells lacking BRCA2 at sites of TRCs that have the potential for forming R-loops. It is possible that here we capture only a subset of the lesions that persist into mitosis and that other forms of DNA damage arise that are repaired by other mechanisms. Monitoring the emergence and repair of DNA damage throughout the entire S-phase progression is required to address this problem.

Moreover, detection of MiDAS depends on the ability of cells to enter mitosis. It is conceivable that high levels of DNA damage may lead to checkpoint activation and prevent mitotic entry, which could reduce the number of MiDAS events observed in both BRCA2-deficient or aphidicolin-treated cells. The latter exhibit a lower rate of mitotic entry than untreated controls, suggesting that the high levels of replication stress induced by aphidicolin arrest the cell cycle before progression into mitosis.

## STAR★Methods

### Key resources table

**Table:** 

REAGENT or RESOURCE	SOURCE	IDENTIFIER
**Antibodies**		

RAD51 primary antibody (rabbit)	BioAcademia	Cat# 70-002
53BP1 primary antibody (rabbit)	Novus Biologicals	Cat# NB100-304; RRID:AB_10003037
53BP1 primary antibody (mouse)	Thanos Halazonetis (Schultz et al., 2000)	N/A
H2AX Phospho S139 primary antibody (rabbit)	Cell Signaling	Cat# 9718; RRID:AB_2118009
Histone H3 Phospho S10 primary antibody (mouse)	Cell Signaling	Cat# 9701; RRID:AB_331535
BRCA2 primary antibody (mouse)	Calbiochem	Cat# OP95; RRID:AB_2067762
SMC1 primary antibody (rabbit)	Bethyl Laboratories	Cat# A300-055A; RRID:AB_2192467
GAPDH primary antibody (mouse)	Novus Biologicals	Cat# NB600-502; RRID:AB_10077682
FLAG primary antibody (mouse)	Agilent Technologies (Stratagene)	Cat# 200470-21
KAP1 Phospho S824 primary antibody (rabbit)	Bethyl Laboratories	Cat# A300-767A; RRID:AB_669740
KAP1 (rabbit)	Bethyl Laboratories	Cat# A300-274A; RRID:AB_185559
Alexa Fluor 568 goat anti-rabbit	ThermoFisher	Cat# A11036; RRID:AB_10563566
Alexa Fluor 594 goat anti-rabbit	ThermoFisher	Cat # A11012; RRID:AB_2534079
Alexa Fluor 488 goat anti-mouse	ThermoFisher	Cat# A11029; RRID:AB_2534088
Alexa Fluor 488 goat anti-mouse	ThermoFisher	Cat# A10667; RRID:AB_2534057
anti-mouse HRP-conjugated secondary antibody	DAKO	Cat# P0447; RRID:AB_2617137
anti-rabbit HRP-conjugated secondary antibody	DAKO	Cat# P0448; RRID:AB_2617138

**Chemicals, peptides, and recombinant proteins**		

Dulbecco’s modified Eagle’s medium (DMEM)	Sigma-Aldrich	Cat# D5796
Tet system approved fetal bovine serum	Takara Bio	Cat# 631106
Doxycycline	Sigma-Aldrich	Cat# D9891
Tetracycline	Sigma-Aldrich	Cat# T7660
Thymidine	Sigma-Aldrich	Cat# T1895
RO-3306	Sigma-Aldrich	Cat# SML0569
Hydroxyurea	Sigma-Aldrich	Cat# H8627
Nocodazole	Sigma-Aldrich	Cat# M1404
EdU	ThermoFisher	Cat# C10340
Aphidicolin	Sigma-Aldrich	Cat# A0781
DRB	Sigma-Aldrich	Cat# D1916
EU	ThermoFisher	Cat# E10345
INTERFERin (transfection reagent)	Polyplus	Cat# 409-01
Dharmafect1 (transfection reagent)	Horizon Discovery	Cat# T-2001-03
Azide-PEG(3+3)-S-S-biotin	Jena Biosciences	Cat# CLK-A2112-10
Dynabeads MyOne streptavidin C1	ThermoFisher	Cat# 65001
β-mercaptoethanol	Sigma-Aldrich	Cat# M6250
TRIzol reagent	ThermoFisher	Cat# 15596026
HiMark Pre-stained Protein Standard	ThermoFisher	Cat# LC5699
ProLong Gold Antifade Mountant	ThermoFisher	Cat# P36930
DAPI	ThermoFisher	Cat# D1306
Mowiol 4-88	Sigma-Aldrich	Cat# 475904
Propidium iodide solution	Sigma-Aldrich	Cat# P4864
PureLink RNase A	ThermoFisher	Cat# 12091021

**Critical commercial assays**		

Click-iT EdU Alexa Fluor 647 Cell Proliferation Kit for Imaging	ThermoFisher	Cat# C10340
Click-iT Nascent RNA Capture Kit	ThermoFisher	Cat# C10365
Click-iT EdU Alexa Fluor 647 Flow Cytometry Assay Kit	ThermoFisher	Cat# C10424
TruSeq ChIP Library Preparation Kit	Illumina	Cat# IP-202-1012
TruSeq Stranded Total RNA with Ribo-Zero Gold	Illumina	Cat# RS-122-2301

**Deposited data**		

DRIP-seq data (HeLa cells)	Hamperl et al., 2017	GEO: GSM2452072
qDRIP-seq data (HeLa cells)	Crossley et al., 2020	GEO: GSE134084
OK-seq data (RFD in HeLa cells)	Petryk et al., 2016	https://github.com/CL-CHEN-Lab/OK-Seq/tree/master/published_results/HeLa
EdU-seq BRCA2-deficient H1299	Dagg et al., 2021	GEO: GSM4650337
MiDAS-seq data	This paper	GEO: GSE196163
EU-seq data	This paper	GEO: GSE196163

**Experimental models: Cell lines**		

H1299 +shBRCA2^DOX^ cells (male origin)	Zimmer et al., 2016	N/A
HeLa cells (female origin)	ATCC	Cat# CCL-2
HeLa +RNaseH1-FLAG^DOX^ (female origin)	Sollier et al., 2014	N/A
U2OS CyclinE (tet OFF) RAD52^+/+^ (female origin)	Sotiriou et al., 2016	N/A
U2OS CyclinE (tet OFF) RAD52^-/-^ (female origin)	Sotiriou et al., 2016	N/A
U2OS T-REx GFP-RnaseH1^D210N^ (female origin)	Teloni et al., 2019	N/A

**Oligonucleotides**		

siGENOME *BRCA2* siRNA (SMARTpool)	Horizon Discovery	Cat# M-003462-01
ON-TARGETplus *RAD52* siRNA (SMARTpool)	Horizon Discovery	Cat# L-011760-00-0005
*BRCA2* siRNA	Qiagen	Cat# SI02653434
AllStar negative control	Qiagen	Cat# 1027281
*TP53* siRNA	Dharmacon	Cat# GEHCU-000146
ON-TARGETplus non-targeting control pool	Horizon Discovery	Cat# D-001810-10-05

**Software and algorithms**		

GraphPad Prism	GraphPad	https://www.graphpad.com/
Olympus ScanR Image Analysis Software 3.0.0	Olympus	https://www.olympus-lifescience.com/en/microscopes/inverted/scanr/
TIBCO Spotfire	TIBCO Software	https://www.tibco.com/products/tibco- spotfire
Fiji	Schindelin et al., 2012	https://fiji.sc/
Bedtools	Quinlan and Hall, 2010	https://bedtools.readthedocs.io/en/latest/
DeepTools	Ramírez et al., 2016	https://deeptools.readthedocs.io/en/develop/
IGV-Web app version 1.7.0	Robinson et al., 2011	https://software.broadinstitute.org/software/igv/
EdU-seq processing and plotting	Macheret and Halazonetis, 2018	N/A
FlowJo	BD Biosciences	https://www.flowjo.com/

### Resource availability

#### Lead contact

Further information and requests for resources and reagents should be directed to and will be fulfilled by the lead contact, Madalena Tarsounas (madalena.tarsounas@oncology.ox.ac.uk).

#### Materials availability

This study did not generate new unique reagents.

### Experimental model and subject details

#### Cell lines and growth conditions

All cell lines were grown at 37˚C, 5% CO_2_, under humidified atmosphere and sterile conditions. Cells were routinely tested for the absence of mycoplasma contamination.

Human non-small-cell lung carcinoma H1299 cells (male) carrying a doxycycline (DOX)-inducible shRNA against BRCA2 (H1299+sh*BRCA2*^DOX^) were grown in Dulbecco’s Modified Eagle’s Medium (DMEM) supplemented with 10% Tet system approved foetal bovine serum (FBS) (Takara Bio) as previously described (Zimmer et al., 2016). Expression of shRNA against BRCA2 was induced by adding 2 μg/mL DOX (Sigma-Aldrich) in the growth medium. This cell line was authenticated using the ATCC STR Profiling Service.

Human HeLa cells (female; ATCC, cat# CCL-2) were maintained in DMEM supplemented with 10% fetal bovine serum and penicillin/streptomycin/glutamine.

Human HeLa cells carrying DOX-inducible cassette controlling expression of FLAG-tagged RNase H1 (Sollier et al., 2014) were maintained in DMEM supplemented with 10% Tet system approved foetal bovine serum (FBS) (Takara Bio). Expression was induced by adding 2 μg/mL DOX (Sigma-Aldrich) in the growth medium for 18 hours.

Human RAD52^+/+^ and RAD52^-/-^ U2OS Cyclin E (tet OFF) cells (female; Sotiriou et al., 2016) were grown in DMEM supplemented with 10% fetal bovine serum, and with 400 μg/mL G418, 1 μg/mL puromycin and 2 μg/mL tetracycline (Sigma-Aldrich).

Human U2OS T-REx cells carrying a DOX-inducible cassette controlling expression of catalytically inactive GFP-RNaseH1^D210N^ (Teloni et al., 2019) were grown in DMEM supplemented with 10% Tet system approved FBS (Takara Bio), and with 1 μg/mL puromycin and 50 μg/mL hygromycin. Expression was induced by adding 1 ng/mL DOX (Sigma-Aldrich) in the growth medium for 24 hours.

### Method details

#### MiDAS-seq

H1299+sh*BRCA2*^DOX^ cells were seeded in order to reach 70-80% confluence at the end of the experiment (1.5-2 million cells per T175 flask, 10-25 flasks per condition) and DOX (Sigma-Aldrich) was added where indicated. Cells were synchronized at the G1/S transition with 1.5 mM thymidine (Sigma-Aldrich) for 16 hours. To detect MiDAS under untreated conditions, cells were washed three times with PBS and released in fresh medium containing 6 μM RO-3306 (Sigma-Aldrich) for 10.5 hours. To detect aphidicolin-induced MiDAS, cells were washed three times with PBS and released in fresh medium containing 6 μM RO-3306 (Sigma-Aldrich) and 0.2 μM aphidicolin (Sigma-Aldrich) for 17.5 hours. For both protocols, cells were then washed three times with warm medium and released in medium containing 100 ng/mL nocodazole (Sigma-Aldrich) and 10 μM EdU (ThermoFisher). To suppress replication in S-phase cells that could potentially contaminate the mitotic shake-off 2 mM HU (Sigma-Aldrich) was added during the final 3 hours. Mitotic cells were collected by mitotic shake-off, fixed with 90% ice-cold methanol and stored at -20°C until processed for isolation of EdU-labelled DNA.

#### Isolation and sequencing of EdU-labelled DNA

Cells were washed with PBS and permeabilized with 0.2% Triton-X in PBS. A cleavable biotin linker (Azide-PEG(3+3)-S-S-biotin, Jena Biosciences) was attached to the EdU-labelled DNA using reagents from the Click-iT EdU Flow Cytometry Assay Kit (ThermoFisher). DNA was isolated by phenol-chloroform extraction, precipitated in ethanol, resuspended in TE buffer, and sonicated to 100-500 nucleotide-long fragments. Dynabeads MyOne streptavidin C1 (ThermoFisher) were used to capture EdU-labelled DNA fragments. Beads were washed three times, resuspended in 1X Binding and Washing Buffer (5 mM Tris-HCl, pH 7.5, 0.5 mM EDTA, 1 M NaCl, 0.5% Tween 20) containing the sonicated DNA and incubated on a rotating wheel for 15 min at room temperature. Beads were washed three times with 1X Binding and Washing Buffer and once with Tris-EDTA buffer. EdU-labelled DNA fragments were eluted with 2% β-mercaptoethanol (Sigma-Aldrich) in Tris-EDTA buffer for 1 hour at room temperature. Purified EdU-labelled DNA was used for library preparation (TruSeq ChIP Library Preparation Kit, Illumina) and high-throughput 100-base-pair single-end sequencing was performed on Illumina Hi-Seq 4000 sequencer.

#### EU-seq

Cells were synchronized at the G1/S transition with 1.5 mM thymidine (Sigma-Aldrich) for 16 hours, washed three times with PBS, released in fresh medium for 100 min or 200 min and labelled with 0.5 mM EU (ThermoFisher) for the final 20 min. RNA was then extracted and purified using TRIzol (ThermoFisher) and isopropanol precipitation. A cleavable biotin linker (Azide-PEG(3+3)-S-S-biotin, Jena Biosciences) was attached to the EU-labelled RNA using reagents from the Click-iT Nascent RNA Capture Kit (ThermoFisher). Dynabeads MyOne streptavidin C1 (ThermoFisher) were used to capture the EU-labelled RNA. Beads were washed three times with 1X Binding and Washing Buffer (5 mM Tris-HCl pH 7.5, 0.5 mM EDTA, 1 M NaCL, 0.5% Tween-20), two times with 0.1 M NaOH, 0.05 M NaCl for 2 min and two times with 0.1 M NaCl for 2 min. The RNA was heated at 70°C, cooled on ice and incubated with beads in 2X Binding and Washing Buffer DNA on a rotating wheel for 30 min at room temperature. Beads were washed three times with 1X Binding and Washing Buffer and once with RNase-free water. EU-labelled RNA was eluted with 2% β-mercaptoethanol (Sigma-Aldrich) for 1 hour at room temperature. Purified EU-labelled RNA was used for library preparation by TruSeq Stranded Total RNA with Ribo-Zero Gold (Illumina), omitting the ribo-depletion step, and high-throughput 100-base-pair single-end sequencing was performed on an Illumina Hi-Seq 4000 sequencer.

#### EdU-seq (MiDAS-seq) and EU-seq data processing

Sequencing reads were aligned to the masked human genome assembly (GRCh37/hg19) using the Burrows-Wheeler Aligner software and reads with a quality score below 60 were removed. Previously described scripts were used to assign the aligned reads to 10 kb genomic bins and to calculate sigma (σ) values as the normalized number of reads per bin divided by its standard deviation (Macheret and Halazonetis, 2018). Sigma values were used to identify MiDAS sites (peaks) by searching local maxima (Methods S1). MiDAS peaks were validated manually and subsequently classified as single- or multiple-peaks. Sigma values at individual sites were plotted using previously described scripts (Macheret and Halazonetis, 2018). Sigma values across whole chromosomes were plotted using custom R scripts.

#### qDRIP processing and peak calling

Strand-specific qDRIP data for untreated HeLa cells were downloaded from Sequence Read Archive (SRR10916579 and SRR10916580). Raw data was trimmed and aligned using Cutadapt as described in Crossley et al., (2020). Trimmed reads were aligned to the masked human genome assembly (GRCh37/hg19) using the Burrows-Wheeler Aligner software. Properly paired reads were filtered and sorted using SAMtools. For peak calling, the MACS2 algorithm was used with default broad peak settings for paired-end data. Strand-specific files from the aligned BAM files were obtained using SAMtools.

#### Average sequencing plots and heatmaps

The “bamCoverage” function of deepTools (Ramírez et al., 2016) was used to generate BigWig files using BAM files aligned as described for EdU-seq, EU-seq or qDRIP data processing. Bin size for MiDAS-seq data: 10 kb; EU-seq data: 50 bp; qDRIP-seq: 50: bp. The “computeMatrix” and “plotHeatmap” functions of deepTools were used to generate average plots and heatmaps across multiple regions. BigWig files were used to plot individual sites using the IGV-Web app version 1.7.0 (Robinson et al., 2011).

#### Assignment of replication timing

REPLI-seq data were previously generated in asynchronous U2OS cells (Macheret and Halazonetis, 2018). The Bedtools (Quinlan and Hall, 2010) “intersect” function was used to assign the replication timing of MiDAS peak bin identified by peak calling.

#### Analysis of genic/intergenic regions

A list of all protein-coding genes with HGNC symbol IDs and their position in the genome was obtained from Ensembl BioMart (http://grch37.ensembl.org/biomart/martview/) and downstream analyses were done using the Bedtools “intersect” function. MiDAS sites were classified as genic if at least one gene was found ±50 kb from the MiDAS peak. For the identification of genes and gene size at MiDAS sites, all genes ±50 kb of a MiDAS peak were considered.

#### Analysis of origin proximity

The Bedtools “closest” function was used to calculate the distance between each MiDAS site and its nearest replication origin identified in BRCA2-deficient H1299 cells by EdUseq-HU 20 hours after release from mitosis (Dagg et al., 2021).

#### Random early S-phase and R-loop prone gene set selection

A list of genomic bins (10 kb) replicating in early S-phase was generated based on REPLI-seq data. Genomic bins within 50 bins of each MiDAS peak were excluded from the list of bins replicating in early S-phase. Next, for each genomic bin, a ±50 kb region was defined and classified as R-loop prone if at least one qDRIP peak was found within this region. The number of qDRIP peaks and genes within each of these regions were obtained using Bedtools “intersect” function. Finally, random sampling without replacement was performed to select 10 different sets of 150 early-replicating, R-loop prone, non-MiDAS genomic regions.

#### EU-seq quantification

Mapped reads were summarized using featureCounts version 2.0.3 with flags -s 2 -t gene -M and GRCh37/hg19 annotation file obtained from GENCODE. Features were further annotated using org.Hs.eg.db version 3.14.0 R package to obtain entrez ID and gene symbol. Features with no entrez ID were removed. Values for transcript per million (TPM) and log_10_(TPM+1) were calculated for each gene within MiDAS sites or control sites.

#### Analysis of G-quadruplex density

The number of G-quadruplexes (mapped in primary human B lymphocytes in the presence of pyridostatin; Chambers et al., 2015) across a 110-kb region for each MiDAS and control sites was determined using the Bedtools “intersect -c” function and expressed as the number of G-quadruplexes per kilobase.

#### Analysis of structural variants

Rearrangements were identified in *BRCA2* wild type (*n* =4 69) and in *BRCA2*-mutated (*n* = 39) breast cancer tumors (Nik-Zainal et al., 2016). The Bedtools “intersect” function and custom python scripts were used to assess the presence of rearrangements within ±200 kb from each MiDAS peak. For each MiDAS site, the percentage of tumors with at least one rearrangement is reported.

#### Quantitative Image-Based Cytometry (QIBC)

Cells were grown on coverslips and treated as indicated. Cells were then washed with PBS, fixed with 4% paraformaldehyde in PBS for 15 min at room temperature, washed with PBS, permeabilized with 0.2% Triton X-100 in PBS for 5 min at room temperature and washed three times with PBS. Coverslips were blocked in antibody diluent buffer (DMEM + 10% FBS, 0.02 μM filtered) for 30 min at room temperature. The Click-iT EdU Alexa Fluor 647 Cell Proliferation Kit for Imaging (ThermoFisher) was used for EdU detection, according to the manufacturer’s instructions. Next, the coverslips were washed with antibody diluent buffer and incubated with anti-RAD51 (rabbit, BioAcademia, 1:1000) or anti-53BP1 (rabbit, Novus Biologicals, 1:5000) primary antibodies for 2 hours at room temperature in the dark, washed three times with PBS and incubated with secondary antibodies (Alexa Fluor 568 goat anti-rabbit, ThermoFisher, 1:500) for 1 hour at room temperature in the dark. Coverslips were washed once with PBS, incubated with 4’,6-Diamidino-2-Phenylindole Dihydrochloride (DAPI, 0.5 μg/mL, ThermoFisher) in PBS for 10 min at room temperature in the dark, washed three times with PBS and rinsed in Milli-Q water before being mounted on slides using Mowiol-based mounting media.

QIBC experiments for detection of RAD51 or 53BP1 foci were performed on an Olympus ScanR inverted microscope system equipped with IX83 inverted motorized frame with Z-drift control, Semrock DAPI/FITC/Cy3/Cy5 Quad LED filter set, sCMOS Hamamatsu Orca Fusion B Camera (Pixel size on chip 6.5 μm, Array size, 2304 x 2304 pixels or 5.3M pixels, FOV 14.976 mm x 14.976 mm). An Olympus UPLXAPO 20x, NA 0.80, WD 0.6 mm, Air/Dry objective was used. Light sources were Lumencor SPECTRA X Light Engine Independent LEDs (Violet 395/25 295 mW, Yellow 575/25 310 mW, Red 640/30 231 mW) used at 100% power. Identical exposure times were used for all samples within one experiment. For each sample, a total of 2000-6000 nuclei were captured using the 20x objective, under non-saturating conditions. Images were captured using SCANR Acquistion software (Version 3.2.0).

Images were analyzed using the Olympus ScanR Image Analysis Software (Version 3.2.0). A virtual mask was applied for RAD51 and 53BP1. Segmentation of nuclei and foci was performed using the in-built object detection module based on intensity and size inclusion criteria. All downstream analyses were performed using TIBCO Spotfire software (Version 16.6.0). Fluorescence intensities of the segmented nuclei and foci are depicted as arbitrary units. Cell cycle profiles for each sample were generated using scatter plots of total DAPI intensity plotted against Log mean intensity EdU. Gating strategies for cell cycle analyses are indicated in the figures.

QIBC experiments performed in U2OS T-REx with inducible expression of GFP-RNaseH1^D210N^ were performed as previously described (Teloni et al., 2019).

#### Immunofluorescence

Cells were grown on coverslips, treated as indicated and fixed in 4% paraformaldehyde for 15 min at room temperature. The coverslips were washed twice with PBS, permeabilized for 5 min in 0.2% Triton X-100, followed by 20 min blocking in filtered 1% BSA in PBS at room temperature. Primary antibody incubations were performed at room temperature for 2 hours. Secondary antibody incubations were performed at room temperature for 30 min. The coverslips were washed twice with PBS, incubated with DAPI (0.5 μg/mL, ThermoFisher) in PBS for 10 min at room temperature, washed three times with PBS and mounted on slides using Mowiol-based mounting media. Primary antibodies used: anti-53BP1 (mouse, 1:10; Schultz et al., 2000); anti-H2AX Phospho S139 (rabbit, Cell Signaling, 1:500). Secondary antibodies used: Alexa Fluor 488 (goat anti-mouse, ThermoFisher, 1:500); Alexa Fluor 594 (goat anti-rabbit, ThermoFisher, 1:500). Images were acquired with a Zeiss Imager M2 AX10 microscope as described in the section describing detection of mitotic EdU foci.

#### Detection of mitotic EdU foci

For detection of mitotic EdU foci in H1299+sh*BRCA2*^DOX^ cells, cells were synchronized at the G1/S transition with 1.5 mM thymidine (Sigma-Aldrich) for 16 hours, washed three times with PBS, released in fresh medium containing 6 μM RO-3306 (Sigma-Aldrich) for 11 hours (in the absence of aphidicolin) or for 17.5 hours (in the presence of 0.2 μM aphidicolin (Sigma-Aldrich)) and treated as indicated. Cells were then washed three times with warm medium and released in medium containing 100 ng/mL nocodazole (Sigma-Aldrich) and 20 μM EdU (ThermoFisher) for 60-90 min before being processed.

For detection of mitotic EdU foci in HeLa cells or in RAD52^+/+^ and RAD52^-/-^ U2OS cells, cells were synchronized at the G1/S transition with 2 mM thymidine (Sigma-Aldrich) for 18 hours, washed four times with PBS and released in fresh medium for 9 hours. Cells were labelled with 20 μM EdU (ThermoFisher) during the final hour, and processed. For detection of mitotic EdU foci upon RNaseH1 overexpression, 2 μg/mL DOX was added 18 hours before release from thymidine block. For detection of mitotic EdU foci in upon ATM inhibition, cells were treated with DMSO or 10 μΜ KU55933 after release from thymidine block.

Cells were fixed and permeabilized with 4% paraformaldehyde, 20 mM HEPES, 10 mM EGTA, 0.2 % Triton X-100, 1 mM MgCl2 for 20 min at room temperature. Coverslips were washed three times with PBS and the Click-iT EdU Alexa Fluor 647 Cell Proliferation Kit for Imaging (ThermoFisher) was used for EdU detection. Coverslips were washed once with PBS, incubated with DAPI (0.5 μg/mL, ThermoFisher) in PBS for 10 min at room temperature, washed three times with PBS and rinsed in distilled water before being mounted on slides using ProLong Gold Antifade Mountant (ThermoFisher) or Mowiol-based mounting media. Samples were viewed with a Leica DMI6000B inverted microscope and fluorescence imaging workstation equipped with HCX PL APO ×100/1.4–0.7 oil objective or with a Zeiss Imager M2 AX10 microscope equipped with ApoTome2 and a Plan-APOCHROMAT x100/1.4 oil immersion objective and using the ZEN3.4 (blue edition) software. Images were analyzed with ZEN3.4 (blue edition) or ImageJ/FIJI software (National Healthcare Institute, USA; (Schindelin et al., 2012).

#### siRNA transfections

Cells were transfected with siRNAs using Dharmafect1 (Horizon Discovery) or INTERFERin (Polyplus) according to manufacturer’s instructions. Briefly, cells were transfected with 40 nM siRNAs by reverse transfection. The next day, the medium was replaced by fresh medium. The following siRNAs were used: si*BRCA2* (Qiagen, SI02653434), siGENOME *BRCA2* SMARTpool (Dharmacon, M-003462-01), si*TP53* (Dharmacon, GCA UCU UAU CCG AGU GGA AUU UU), si*RAD52* SMARTpool (Dharmacon, L-011760-00-0005). AllStars Negative Control siRNA (Qiagen, 1027281) or ON-TARGETplus non-targeting pool siRNA (Dharmacon, D-001810-10-05) were used as control.

#### Flow cytometry analyses

Cells were collected using trypsin and fixed in 90% ice-cold methanol overnight. Cells were permeabilized using a saponin-based buffer from the Click-iT EdU Flow Cytometry Assay Kit (ThermoFisher), washed with 2% FBS in PBS and incubated with anti-Histone H3 Phospho S10 antibody (mouse, Cell Signaling) diluted in 2% FBS in PBS (1:50) for 90 min at room temperature. Cells were washed with 2% FBS in PBS and incubated with Alexa Fluor488 (goat anti-mouse, ThermoFisher) diluted in 2% FBS in PBS (1:200) for 1 hour at room temperature. Finally, cells were washed in 2% FBS in PBS and resuspended in PBS containing 20 μg/mL propidium iodide (Sigma-Aldrich) and 400 μg/mL RNaseA (ThermoFisher). A total of 5,000 to 10,000 events per condition were recorded using a FACS Calibur flow cytometer. Flow cytometry data were analyzed with the FlowJo software.

#### Western blotting

Cells were lysed using loading buffer (0.16 M Tris pH 8, 4% sodium dodecyl sulfate-polyacrylamide (SDS), 20% glycerol, 0.01% bromophenol blue) supplemented with 100 mM dithiothreitol (DTT), and protease and phosphatase inhibitor cocktails (Roche). Samples were sonicated for 3 sec on ice, heated at 70°C for 10 min and centrifuged at >20,000 g for 7 min. The protein concentration was determined using a NanoDrop-1000 spectrophotometer. Equal amounts of protein were loaded on 3-8% Tris-acetate gels (ThermoFisher). Gels were run in Tris-acetate buffer (ThermoFisher) at 100-180 V until the desired separation was achieved. HiMark prestained protein standard (ThermoFisher) were used as molecular weight markers. Protein transfer onto a nitrocellulose membrane was run in transfer buffer supplemented with 10% methanol (ThermoFisher) at 30 V for 100 min at room temperature. The membranes were subsequently blocked in 5% skimmed milk dissolved in 0.05% Tween 20 in PBS (PBST). Membranes were incubated with primary antibodies diluted in 2% bovine serum albumin and 0.05% azide in PBST over-night at 4°C and with horseradish peroxidase (HRP)-conjugated secondary antibodies diluted in 5% skimmed milk in PBST for 1 hour at room-temperature. Detection was achieved by enhanced chemiluminescence detected on X-ray films.

### Quantification and statistical analysis

#### Statistical analysis

For MiDAS-seq, we performed at least two independent experiments for each genotype under similar conditions. For EU-seq, we performed the experiment at two different timepoints (100 minutes and 200 minutes after release from thymidine block). For the analysis of sequencing data, the number (*n*) of genomic regions analyzed from one experiment is indicated in the figure legend. For QIBC data in Figures 3C, 6A, S5B, and S5D and for FACS data in Figures S1B–S1D, representative single cell data of cell cohorts are shown as two-dimensional cell cycle-resolved scatterplots. For all other figures, the number (*n*) of independent experiments, of analyzed genomic sites or tumors, details of the plotted data and statistical tests are indicated in the figure legend. Statistical analyses were performed with GraphPad Prism using one-way ANOVA followed by a Tukey test, unpaired two-tailed *t*-test or a two-tailed Mann–Whitney test as indicated in the figure legends. Statistical significance was defined as *P* ≤ 0.05.

## Data Availability

• Sequencing data have been deposited at GEO repository and are publicly available as of the date of publication. Accession numbers are listed in the key resources table. This paper analyses existing, publicly available data. These accession numbers for the datasets are listed in the key resources table. All other data reported in this paper will be shared by the lead contact upon request. • All original code is available in this paper’s supplemental information. • Any additional information required to reanalyze the data reported in this paper is available from the lead contact upon request.

## Supplemental information

Document S1. Figures S1–S6

Methods S1. Peak calling script for MiDAS-seq, related to STAR Methods

Table S1 List of MiDAS sites in BRCA2-deficient (−BRCA2) H1299 human cells, related to Figure 1

Table S2 List of MiDAS sites in aphidicolin-treated BRCA2-proficient (+BRCA2+APH) H1299 human cells, related to Figure 1

Table S3 DRIP signal measured in unperturbed HeLa cells at MiDAS sites identified in BRCA2-deficient (−BRCA2) H1299 cells, related to Figure 5

Table S4 List of random early-S & R-loop prone sites, related to Figure 5

Document S2. Article plus supplemental information
