# Supplementary material for: Mitotic DNA synthesis is caused by transcription-replication conflicts in BRCA2-deficient cells
Source: Mol Cell. 2022 Sep 15;82(18):3382–3397.e7. doi: 10.1016/j.molcel.2022.07.011 (PMC9631240; doi:10.1016/j.molcel.2022.07.011)
Supplement: Document S1. Figures S1–S6 [file mmc1.pdf]

Molecular Cell, Volume *82*

Supplemental information

Mitotic DNA synthesis is caused by transcription-replication conflicts in BRCA2-deficient cells

Florian J. Groelly, Rebecca A. Dagg, Michalis Petropoulos, Giacomo G. Rossetti, Birbal Prasad, Andreas Panagopoulos, Teressa Paulsen, Angeliki Karamichali, Samuel E. Jones, Fena Ochs, Vasilis S. Dionellis, Emilia Puig Lombardi, Matthieu J. Miossec, Helen Lockstone, Gaëlle Legube, Andrew N. Blackford, Matthias Altmeyer, Thanos D. Halazonetis, and Madalena Tarsounas

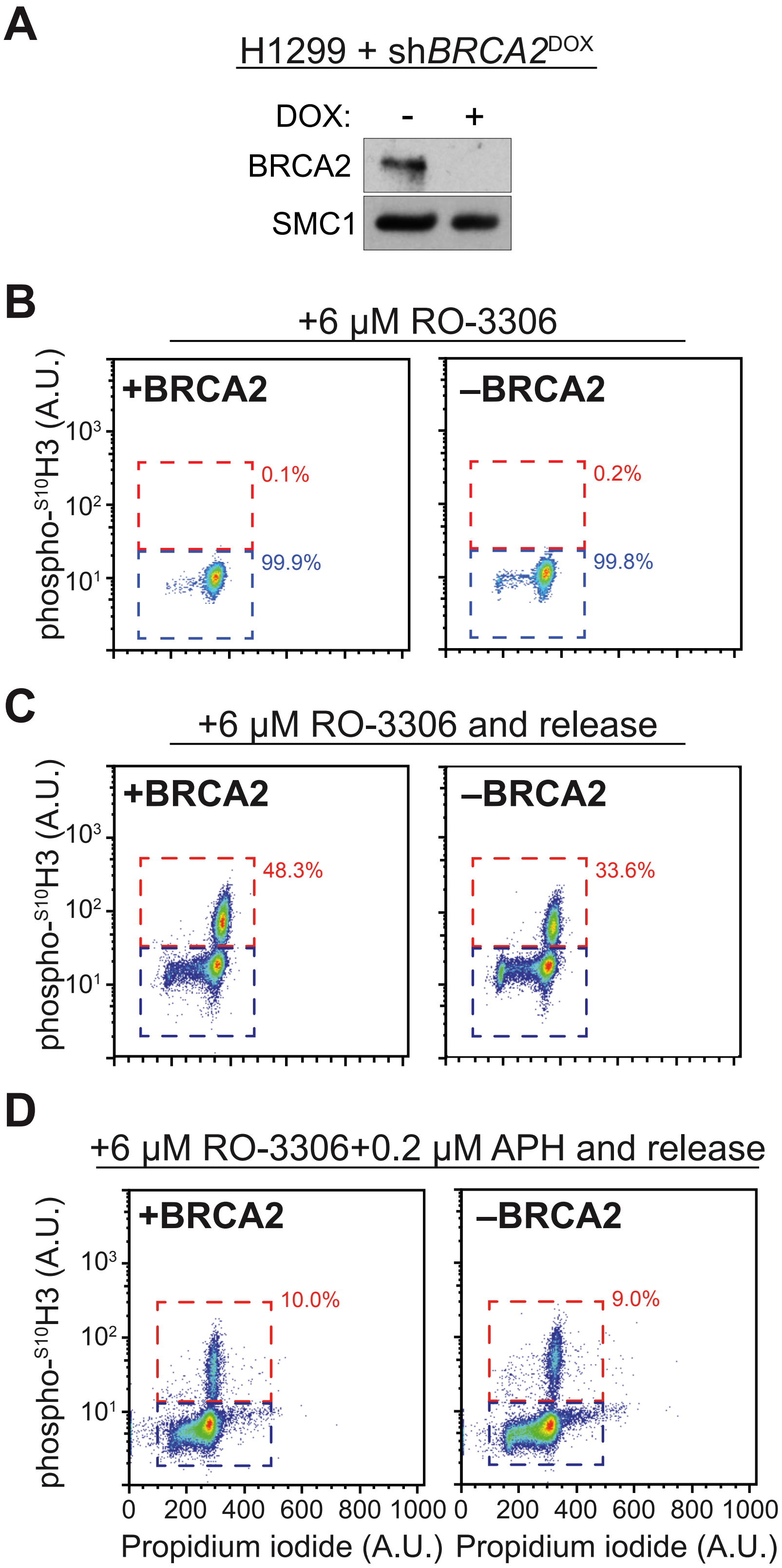
A
H1299 + sh*BRCA2*DOX
DOX: - +
BRCA2
SMC1
B
+6 µM RO-3306
+BRCA2
–BRCA2
0.1%
99.9%
0.2%
99.8%
phospho-S10H3 (A.U.)
C
+6 µM RO-3306 and release
+BRCA2
–BRCA2
48.3%
33.6%
D
+6 µM RO-3306+0.2 µM APH and release
+BRCA2
–BRCA2
10.0%
9.0%
0 200 400 600 800 1000
Propidium iodide (A.U.)

Figure S1. Experimental Conditions for MiDAS-seq in BRCA2-proficient (-DOX) or -deficient (+DOX) H1299 Cells. Related to Figure 1.

(A) Whole-cell extracts of H1299 cells grown in the presence or absence of doxycycline for 48 hours were immunoblotted as shown. SMC1 was used as loading control.

(B,C) BRCA2-proficient (+BRCA2) or -deficient (-BRCA2) H1299 cells grown as in Figure 1A were collected before **(B)** or after **(C)** release from RO-3306 and analyzed by flow cytometry for DNA content and phosphorylated Histone H3 (Serine 10) staining.

(D) Aphidicolin-treated H1299 cells grown as in Figure 1B were analyzed by flow cytometry for DNA content and phosphorylated Histone H3 (^{S10}H3) staining.

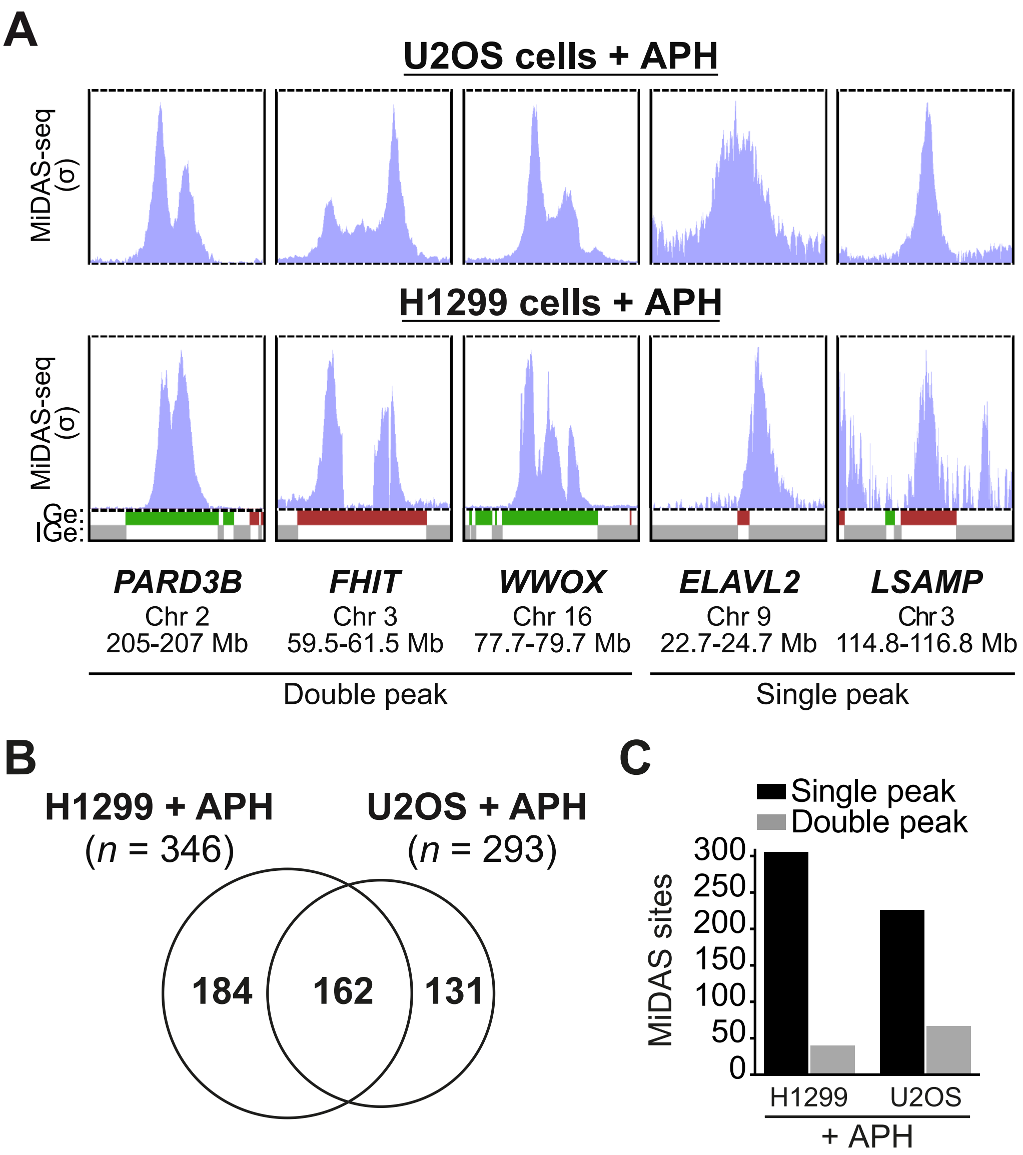
A
U2OS cells + APH
MiDAS-seq (σ)
H1299 cells + APH
MiDAS-seq (σ)
Ge:
IGe:
PARD3B
Chr 2
205-207 Mb
FHIT
Chr 3
59.5-61.5 Mb
WWOX
Chr 16
77.7-79.7 Mb
ELAVL2
Chr 9
22.7-24.7 Mb
LSAMP
Chr 3
114.8-116.8 Mb
Double peak
Single peak
B
H1299 + APH
(n = 346)
U2OS + APH
(n = 293)
184
162
131
C
Single peak
Double peak
MiDAS sites
300
250
200
150
100
50
0
H1299
U2OS
+ APH

Figure S2. Comparison between Aphidicolin-induced MiDAS Sites in U2OS and H1299 Cells. Related to Figure 1.

(A) MiDAS-seq profiles (σ-values) at representative genomic regions in aphidicolin-treated (+APH) U2OS cells (Macheret *et al.*, 2020) and H1299 cells. Ge, genes; IGe, intergenic regions. Bin resolution, 10 kb.

(B) Venn diagram of overlapping MiDAS sites (within ±600 kb) between aphidicolin-treated (+APH) U2OS cells (Macheret *et al.*, 2020) and H1299 cells.

(C) Distribution of single- and double-peak MiDAS sites in aphidicolin-treated (+APH) U2OS cells (Macheret *et al.*, 2020) and H1299 cells.

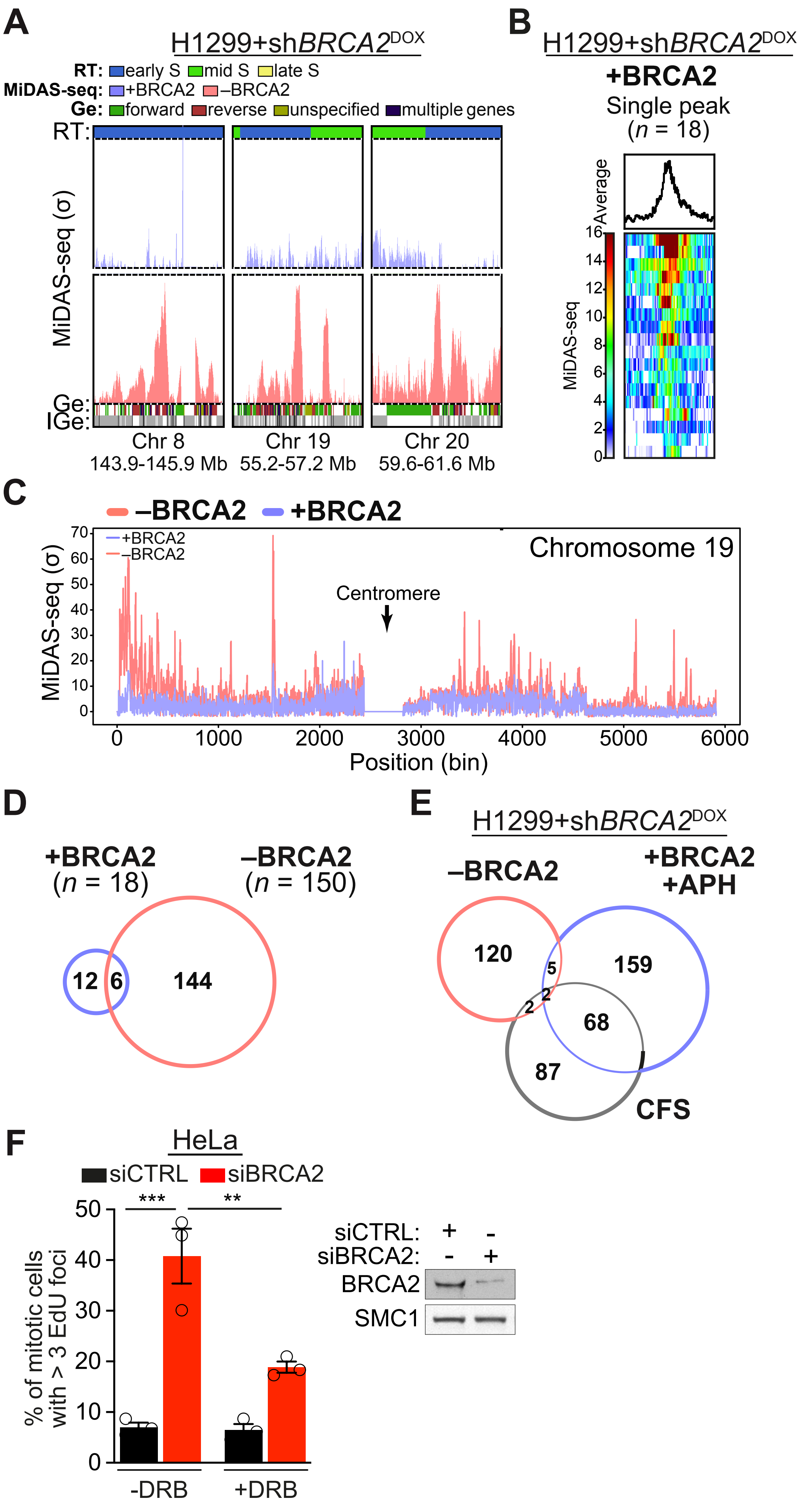
A
H1299+shBRCA2DOX
RT: early S mid S late S
MiDAS-seq: +BRCA2 –BRCA2
Ge: forward reverse unspecified multiple genes
RT:
MiDAS-seq (σ)
Ge:
IGe:
Chr 8
143.9-145.9 Mb
Chr 19
55.2-57.2 Mb
Chr 20
59.6-61.6 Mb
B
H1299+shBRCA2DOX
+BRCA2
Single peak
(n = 18)
Average
MiDAS-seq
16
14
12
10
8
6
4
2
0
C
–BRCA2 +BRCA2
+BRCA2
–BRCA2
Chromosome 19
Centromere
MiDAS-seq (σ)
70
60
50
40
30
20
10
0
0
1000
2000
3000
4000
5000
6000
Position (bin)
D
+BRCA2
(n = 18)
–BRCA2
(n = 150)
12
6
144
E
H1299+shBRCA2DOX
–BRCA2
+BRCA2
+APH
120
5
159
2
2
68
87
CFS
F
HeLa
siCTRL siBRCA2
***
**
% of mitotic cells
with > 3 EdU foci
50
40
30
20
10
0
-DRB
+DRB
siCTRL: + -
siBRCA2: - +
BRCA2
SMC1

Figure S3. MiDAS Induced by BRCA2 Abrogation Requires Transcription. Related to Figures 1, 2 and Figure 4.

(A) MiDAS-seq profiles (σ-values) at representative genomic regions in BRCA2-proficient (+BRCA2; blue) and BRCA2-deficient (-BRCA2; pink) H1299 cells. RT, replication timing; Ge, genes; IGe, intergenic regions. Bin resolution, 10 kb.

(B) Average MiDAS-seq signal across all identified peaks in BRCA2-proficient (+BRCA2) H1299 cells. Span of genomic region, 880 kb.

(C) Whole-chromosome view of MiDAS-seq profiles (σ-values) across chromosome 19 in BRCA2-proficient (+BRCA2; blue) and -deficient (-BRCA2; pink) H1299 cells.

(D) Venn diagram of overlapping MiDAS sites (within ±600 kb) between BRCA2-proficient (+BRCA2) and -deficient (-BRCA2) H1299 cells.

(E) Venn diagram showing overlap between genes found at MiDAS sites in BRCA2-deficient (-BRCA2) H1299 cells, in aphidicolin-treated BRCA2-proficient (+BRCA2 +APH) H1299 cells and within cytogenetically-defined common fragile sites (CFS; (Wilson *et al.*, 2015)).

(F) HeLa cells transfected with the indicated siRNAs were synchronized 48 hours later at the G1/S transition by treatment with 2mM thymidine for 18 hours. Cells were then released for 9 hours in the presence or absence of 75 μM DRB for the first 100 minutes. EdU was added during the final hour and cells were prepared for detection of mitotic EdU foci. Mitotic cells having >3 EdU foci per cell were quantified. Graph and error bars represent the mean and SEM of n = 3 independent experiments. A minimum of 60 cells were analyzed per condition per experiment. P values were calculated using one-way ANOVA followed by a Tukey test. **, $P \leq 0.01$; ***, $P \leq 0.001$. Whole-cell extracts of HeLa cells transfected with indicated siRNAs were immunoblotted as shown. SMC1 was used as loading control.

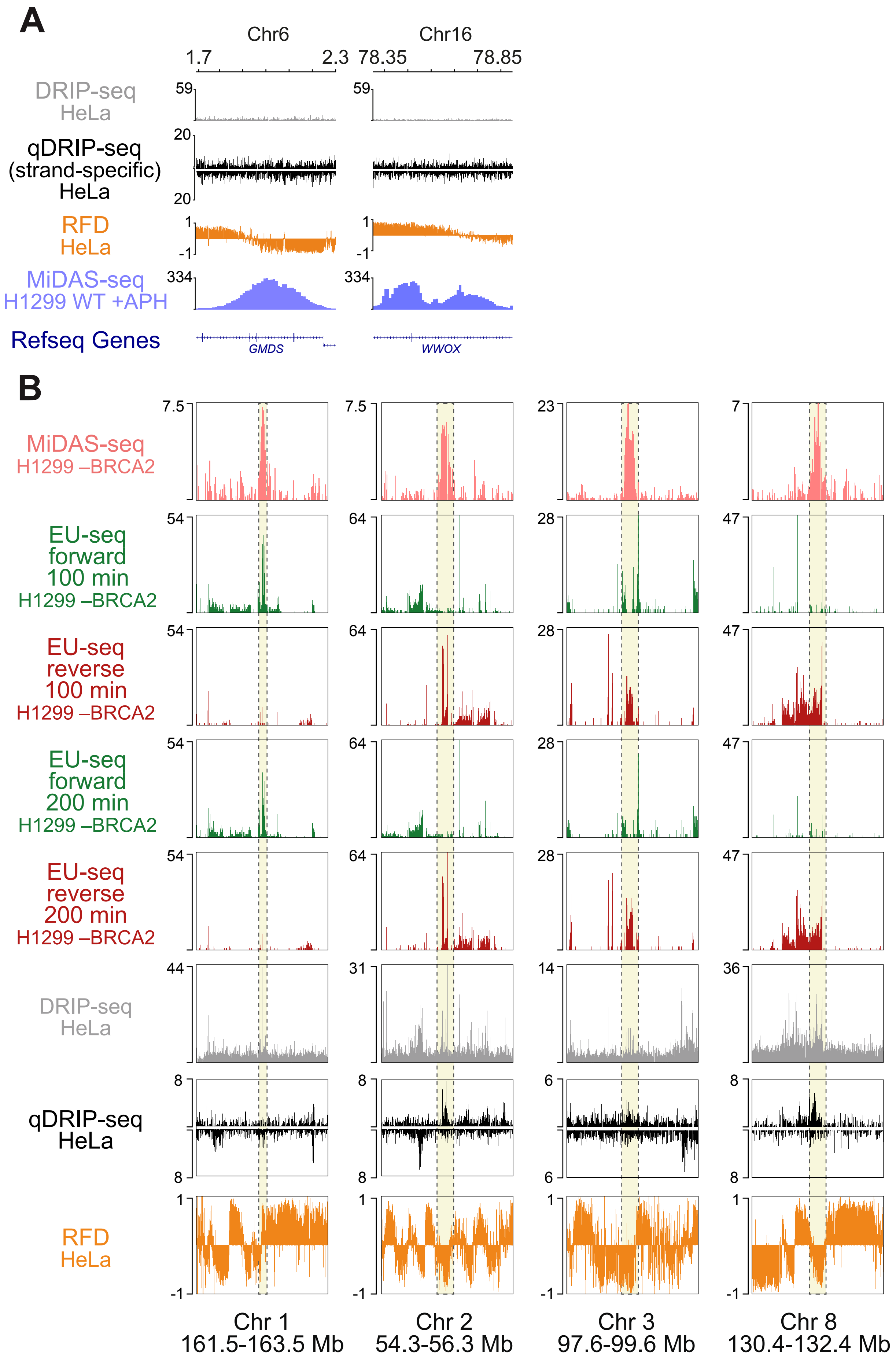
A
Chr6
Chr16
1.7
2.3
78.35
78.85
DRIP-seq
HeLa
qDRIP-seq
(strand-specific)
HeLa
RFD
HeLa
MiDAS-seq
H1299 WT +APH
Refseq Genes
GMDS
WWOX
B
MiDAS-seq
H1299 –BRCA2
EU-seq
forward
100 min
H1299 –BRCA2
EU-seq
reverse
100 min
H1299 –BRCA2
EU-seq
forward
200 min
H1299 –BRCA2
EU-seq
reverse
200 min
H1299 –BRCA2
DRIP-seq
HeLa
qDRIP-seq
HeLa
RFD
HeLa
Chr 1
161.5-163.5 Mb
Chr 2
54.3-56.3 Mb
Chr 3
97.6-99.6 Mb
Chr 8
130.4-132.4 Mb

Figure S4. Co-directional Transcription and R-loops are found at MiDAS sites. Related to Figure 5.

(A) Representative genomic regions for DRIP-seq or qDRIP-seq analyses performed in asynchronous HeLa cells (Hamperl *et al.*, 2017; Crossley *et al.*, 2020), RFD analysis performed in HeLa cells seq (Petryk *et al.*, 2016) and MiDAS-seq analysis performed in aphidicolin-treated BRCA2-proficient (+BRCA2 +APH) H1299 cells.

(B) Representative genomic regions (also shown in Figure 3A) of MiDAS-seq, EU-seq, DRIP-seq, qDRIP-seq and RFD analyses performed in the cell lines indicated. EU-seq was performed 100 or 200 minutes after release from thymidine block.

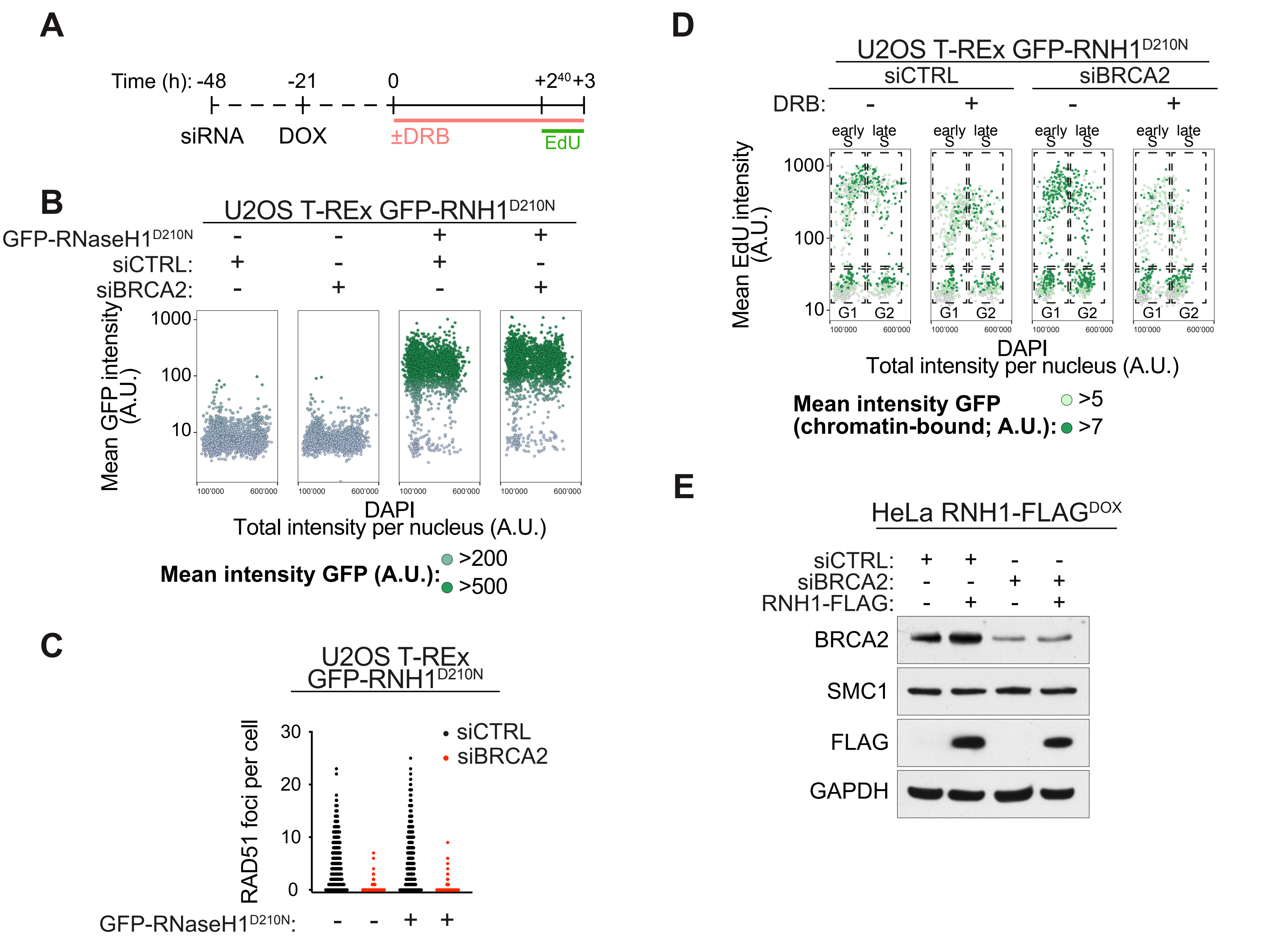
A
Time (h): -48 -21 0 +2 40 +3
siRNA
DOX
±DRB
EdU
B
U2OS T-REx GFP-RNH1 D210N
GFP-RNaseH1 D210N: - - + +
siCTRL: + - + -
siBRCA2: - + - +
Mean GFP intensity (A.U.)
1000
100
10
100'000
600'000
DAPI
Total intensity per nucleus (A.U.)
Mean intensity GFP (A.U.): >200 >500
C
U2OS T-REx GFP-RNH1 D210N
RAD51 foci per cell
30
20
10
0
siCTRL
siBRCA2
GFP-RNaseH1 D210N: - - + +
D
U2OS T-REx GFP-RNH1 D210N
siCTRL
siBRCA2
DRB: - + - +
early S
late S
G1
G2
Mean EdU intensity (A.U.)
1000
100
10
100'000
600'000
DAPI
Total intensity per nucleus (A.U.)
Mean intensity GFP (chromatin-bound; A.U.): >5 >7
E
HeLa RNH1-FLAG DOX
siCTRL: + + - -
siBRCA2: - - + +
RNH1-FLAG: - + - +
BRCA2
SMC1
FLAG
GAPDH

Figure S5. BRCA2-depleted cells accumulate RNA-DNA hybrids. Related to Figure 5.

(A) Experimental timeline for detection of RNA-DNA hybrids in inducible U2OS T-REx GFP-RNaseH1^{D210N} cells transfected with control or BRCA2 siRNAs and treated or not with DRB for the final 3 hours.

(B) QIBC scatter plots showing DNA content versus GFP mean intensity in fixed (non-pre-extracted) cells, with the latter indicated in color scale.

(C) Quantification of RAD51 foci per cell was included as a control for *BRCA2* knockdown.

(D) Chromatin-bound GFP-RNaseH1^{D210N} levels were analyzed in a cell cycle-dependent manner in cells pre-extracted on ice with 0.2% Triton X-100 in PBS. QIBC scatter plots of DNA content versus EdU mean intensity show GFP mean intensity in color scale. Boxes indicate cell populations used for quantification.

(E) Whole-cell extracts of HeLa cells expressing a DOX-inducible, FLAG-tagged RNase H (FLAG-RNH1) treated as in Figure 5F were immunoblotted as shown. SMC1 and GAPDH were used as loading control.

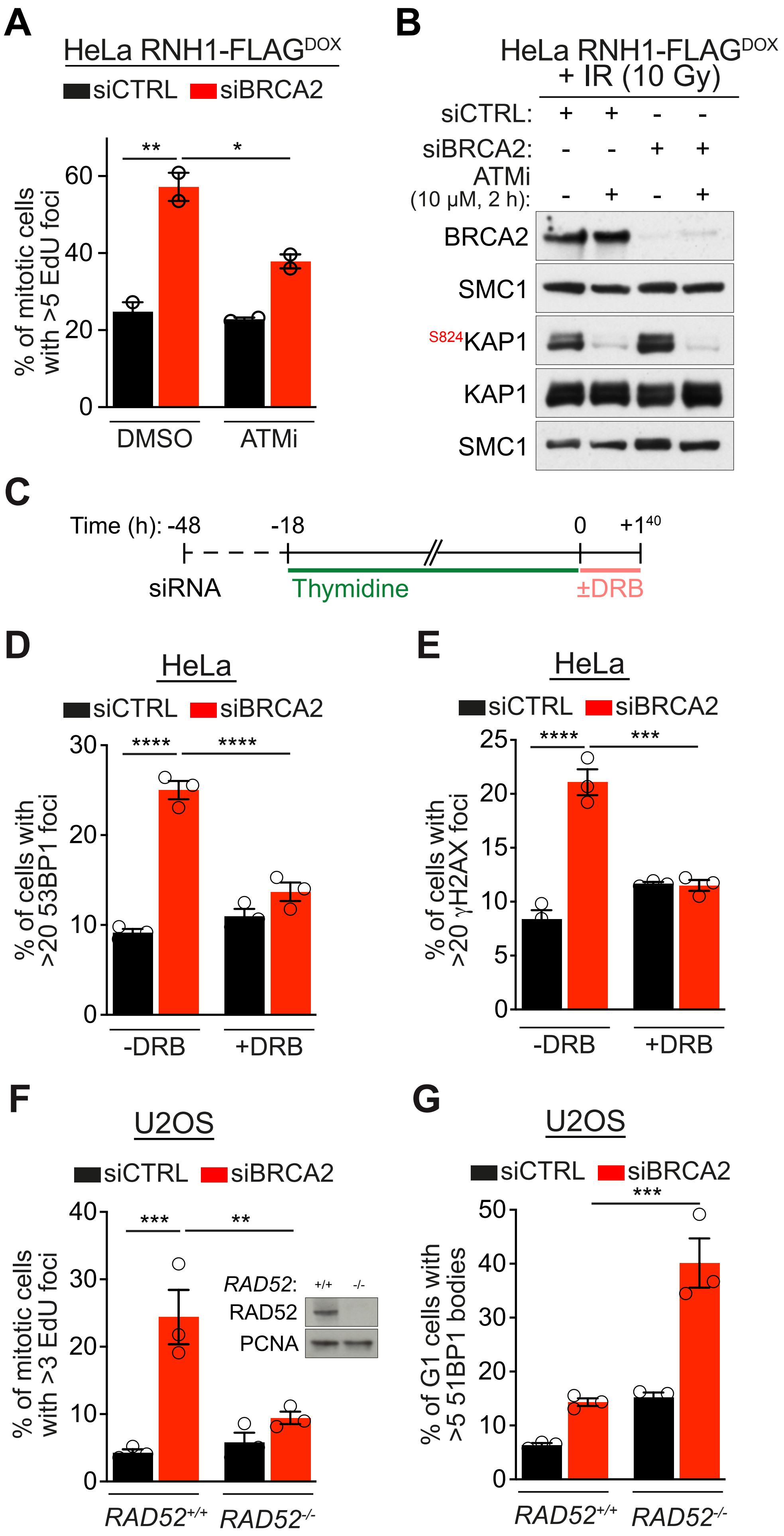
A
HeLa RNH1-FLAG^DOX
siCTRL siBRCA2
% of mitotic cells with >5 EdU foci
0
20
40
60
** *
DMSO ATMi
B
HeLa RNH1-FLAG^DOX + IR (10 Gy)
siCTRL: + + - -
siBRCA2: - - + +
ATMi (10 µM, 2 h): - + - +
BRCA2
SMC1
S824 KAP1
KAP1
SMC1
C
Time (h): -48 -18 0 +1 40
siRNA
Thymidine
±DRB
D
HeLa
siCTRL siBRCA2
% of cells with >20 53BP1 foci
0
10
20
30
**** ****
-DRB +DRB
E
HeLa
siCTRL siBRCA2
% of cells with >20 γH2AX foci
0
5
10
15
20
25
**** ***
-DRB +DRB
F
U2OS
siCTRL siBRCA2
% of mitotic cells with >3 EdU foci
0
10
20
30
40
*** **
RAD52: +/+ -/-
RAD52
PCNA
RAD52+/+ RAD52-/-
G
U2OS
siCTRL siBRCA2
% of G1 cells with >5 51BP1 bodies
0
10
20
30
40
50
***
RAD52+/+ RAD52-/-

Figure S6. MiDAS Induced by BRCA2 Abrogation Requires RAD52. Related to Figure 6.

(A) HeLa cells transfected with the indicated siRNAs were synchronized at the G1/S transition by treatment with 2 mM thymidine for 18 hours and released for 9 hours in the presence or absence of 10 μM ATMi (KU55933). EdU was added during the final hour and cells were prepared for detection of mitotic EdU foci. Quantification of mitotic cells with >3 EdU foci. Graph and error bars represent the mean and SEM of $n = 2$ independent experiments. A minimum of 50 cells were analyzed per condition per experiment. P values were calculated using one-way ANOVA followed by a Tukey test. *, $P \leq 0.05$; **, $P \leq 0.01$.

(B) Whole-cell extracts of HeLa cells treated as indicated and collected 2 hours after 10 Gy irradiation and immunoblotted as shown. SMC1 was used as loading control.

(C) Experimental timeline for detection of 53BP1 and γH2AX foci in HeLa cells transfected with control or BRCA2 siRNAs and treated or not with DRB.

(D) Quantification of S-phase cells treated as in **(C)** with >20 53BP1 foci. Graph and error bars represent the mean and SEM of $n = 3$ independent experiments. A minimum of 80 cells were analyzed per condition per experiment. P values were calculated using one-way ANOVA followed by a Tukey test. ****, $P \leq 0.0001$.

(E) Quantification of S-phase cells treated as in **(C)** with >20 γH2AX foci. Graph and error bars represent the mean and SEM of $n = 3$ independent experiments. A minimum of 80 cells were analyzed per condition per experiment. P values were calculated using one-way ANOVA followed by a Tukey test. ***, $P \leq 0.001$; ****, $P \leq 0.0001$.

(F) Quantification of mitotic $RAD52^{+/+}$ or $RAD52^{-/-}$ U2OS cells with >3 EdU foci. Cells were transfected with the indicated siRNA and collected 9 hours after release from single thymidine block. Graph and error bars represent the mean and SEM of $n = 3$ independent experiments. A minimum of 50 cells were analyzed per condition per experiment. P values were calculated using one-way ANOVA. **, $P \leq 0.01$; ***, $P \leq 0.001$. Whole-cell extracts of $RAD52^{+/+}$ or $RAD52^{-/-}$ U2OS cells were immunoblotted as shown. PCNA was used as loading control.

(G) Quantification of G1 $RAD52^{+/+}$ or $RAD52^{-/-}$ U2OS cells with >5 53BP1 nuclear bodies. Cells were transfected with the indicated siRNA and collected 10 hours after release from single thymidine block. Graph and error bars represent the mean and

SEM of $n = 3$ independent experiments. A minimum of 60 cells were analyzed per condition per experiment. P values were calculated using one-way ANOVA followed by a Tukey test. ***, $P \leq 0.001$.
